# Molecular immunopharmacology of traditional Chinese medicine–derived compounds in membranous nephropathy: mechanistic insights into immune aging and kidney essence deficiency

**DOI:** 10.3389/fimmu.2026.1770018

**Published:** 2026-04-22

**Authors:** Sichao Ma, Mingxin Chang, Yabin Cui, Hong ‘an Wang, Yunfan Liu, Shoulin Zhang

**Affiliations:** 1 Department of Nephrology, Affiliated Hospital of Changchun University of Chinese Medicine, Changchun, Jilin, China; 2 Department of Neurology, Affiliated Hospital of Changchun University of Chinese Medicine, Changchun, China; 3 Affiliated Hospital of Changchun University of Chinese Medicine, Changchun, China

**Keywords:** autoimmunity, immunomodulatory compounds, membranous nephropathy, podocyte injury, precision medicine, signaling pathways, traditional Chinese medicine

## Abstract

Membranous nephropathy (MN) is a prototypical immune-mediated glomerular disease characterized by the formation of autoantibodies targeting podocyte antigens, deposition of subepithelial immune complexes, and activation of complement pathways leading to podocyte injury and proteinuria. The discovery of target antigens such as the M-type phospholipase A2 receptor (PLA2R) and thrombospondin type-1 domain-containing 7A (THSD7A) has substantially advanced the understanding of MN immunopathogenesis. Despite these advances, current therapeutic approaches remain limited by incomplete response rates, treatment-related toxicity, and the lack of personalized treatment strategies. Recent studies have highlighted the immunomodulatory potential of bioactive compounds derived from traditional medicinal plants. Several compounds with well-defined molecular structures including icariin, astragaloside IV, catalpol, cordycepin, and Lycium barbarum polysaccharides have demonstrated experimentally validated mechanisms affecting key molecular pathways involved in inflammation and immune regulation. These compounds modulate intracellular signaling networks such as NF-κB, PI3K–Akt signaling, NLRP3 inflammasome activation, AMPK signaling, and oxidative stress pathways, which are closely associated with immune dysregulation and podocyte injury in MN. In parallel, advances in systems biology and precision medicine are transforming the investigation of immune-mediated kidney diseases. Multi-omics technologies, biomarker discovery, artificial intelligence–assisted disease classification, and network pharmacology approaches provide integrative tools for identifying disease mechanisms and therapeutic targets. These analytical frameworks enable the systematic exploration of compound–target interactions and may facilitate the development of personalized treatment strategies for MN. This review integrates current knowledge on the immunopathogenesis of membranous nephropathy with emerging insights into bioactive compounds derived from traditional Chinese medicine (TCM) and modern systems biology approaches. By linking experimentally supported molecular mechanisms with computational and translational research strategies, this work highlights potential avenues for developing innovative immunomodulatory therapies and advancing precision medicine in immune-mediated kidney diseases.

## 1 Introduction

### 1.1 Membranous nephropathy as an immune-mediated kidney disease

Membranous nephropathy (MN) is one of the most common causes of nephrotic syndrome in adults and represents a prototypical autoimmune glomerular disease characterized by immune complex deposition along the glomerular basement membrane (1, 2). Epidemiological studies indicate that MN accounts for approximately 20–30% of adult nephrotic syndrome cases in many regions worldwide, with peak incidence occurring between the fourth and sixth decades of life (3, 4). Although the disease may arise secondary to infections, malignancies, drugs, or systemic autoimmune disorders, nearly 70–80% of cases are classified as primary or idiopathic MN, in which the pathology results from autoimmune responses targeting podocyte antigens (5, 6). A major breakthrough in understanding the autoimmune nature of MN was the discovery of circulating autoantibodies directed against podocyte antigens, particularly the M-type phospholipase A2 receptor (PLA2R), which is expressed on the surface of glomerular podocytes (7, 8). Anti-PLA2R antibodies are detected in approximately 70–80% of patients with primary MN and have become a key diagnostic biomarker as well as a tool for monitoring disease activity and treatment response (9, 10). The binding of these autoantibodies to PLA2R on podocytes leads to the formation of *in situ* immune complexes within the subepithelial space of the glomerular basement membrane (11). These immune complexes trigger activation of the complement cascade, particularly through the lectin and alternative pathways, culminating in the formation of the membrane attack complex (C5b-9) (12, 13). Complement activation is considered a central pathogenic driver in MN because the deposition of C5b-9 on podocyte membranes induces cellular stress responses, cytoskeletal rearrangement, and alterations in podocyte signaling pathways (14, 15). Experimental and clinical studies demonstrate that complement-mediated injury promotes oxidative stress, mitochondrial dysfunction, and inflammatory signaling within podocytes, ultimately leading to structural damage of the glomerular filtration barrier (16). Podocyte injury represents the final common pathway in MN pathogenesis. Podocytes are highly specialized epithelial cells essential for maintaining the integrity of the glomerular filtration barrier through their interdigitating foot processes and slit diaphragm structures (17). In MN, immune complex deposition and complement activation cause podocyte effacement, detachment, and disruption of slit diaphragm proteins such as nephrin and podocin, resulting in increased glomerular permeability and massive proteinuria (18, 19). Persistent immune-mediated injury can therefore drive progressive glomerular damage, chronic inflammation, and eventual decline in renal function if left untreated (8). Collectively, these findings establish MN as a paradigm of antibody-mediated autoimmune kidney disease in which the interplay between autoantibody production, complement activation, and podocyte injury drives disease initiation and progression. Understanding these immunopathogenic mechanisms provides the biological framework for developing targeted immunomodulatory therapies and exploring novel pharmacological strategies derived from bioactive natural compounds (20, 21).

### 1.2 Limitations of current immunosuppressive therapies

The therapeutic management of MN has traditionally relied on immunosuppressive regimens aimed at reducing autoantibody production and controlling immune-mediated glomerular injury (22). Current guideline-recommended therapies primarily include corticosteroids combined with cytotoxic agents such as cyclophosphamide, calcineurin inhibitors (e.g., cyclosporine and tacrolimus), and monoclonal antibody–based B-cell–depleting therapies such as rituximab (23, 24). These treatments target different components of the immune system and have demonstrated efficacy in inducing remission of proteinuria and stabilizing renal function in many patients (25). Cyclophosphamide-based regimens, often administered in combination with corticosteroids in protocols such as the Ponticelli regimen, have historically been considered one of the most effective therapies for high-risk MN patients (26). These regimens reduce autoantibody production by suppressing B-cell proliferation and dampening immune complex formation, thereby limiting complement-mediated podocyte injury (8). However, their clinical use is associated with substantial adverse effects, including myelosuppression, infertility, infections, and an increased long-term risk of malignancy, which restricts their applicability in many patient populations (27). Calcineurin inhibitors (CNIs), including cyclosporine and tacrolimus, represent another widely used class of immunosuppressive drugs in MN management (28). These agents inhibit T-cell activation by blocking calcineurin-dependent signaling and downstream transcription of interleukin-2, thereby suppressing immune responses involved in antibody production (29). In addition to their immunological effects, CNIs exert direct podocyte-stabilizing actions by preserving the cytoskeletal architecture of podocyte foot processes (30). Despite their ability to reduce proteinuria, relapse rates following treatment discontinuation remain high, and long-term use is limited by nephrotoxicity and metabolic complications (31). More recently, biologic therapies targeting B lymphocytes have emerged as an important therapeutic option for MN (32). Rituximab, a monoclonal antibody directed against the CD20 antigen on B cells, induces selective B-cell depletion and reduces the production of pathogenic autoantibodies such as anti-PLA2R antibodies (33, 34). Clinical trials have demonstrated that rituximab can achieve sustained immunological remission and reduce proteinuria in a significant proportion of patients with MN (35). Nevertheless, not all patients respond to B-cell depletion therapy, and some individuals experience delayed responses, incomplete remission, or disease relapse after treatment (36). Beyond therapeutic efficacy, a major limitation of current immunosuppressive strategies lies in their broad systemic immunosuppressive effects. These therapies frequently compromise host immune defenses, increasing susceptibility to infections and impairing immune surveillance mechanisms (37). Furthermore, because MN pathogenesis involves complex interactions among immune cells, complement activation, and podocyte signaling pathways, therapies targeting a single immune component may not fully address the multifactorial nature of the disease (38). Consequently, there is growing interest in identifying novel therapeutic approaches capable of modulating immune responses while minimizing systemic toxicity. Natural products and bioactive compounds derived from traditional medicinal systems have gained attention as potential sources of immunomodulatory agents with multi-target mechanisms of action (16, 39). Several compounds isolated from traditional Chinese medicine (TCM) have been shown in experimental studies to regulate inflammatory signaling pathways, oxidative stress responses, and immune cell function, suggesting potential relevance for the treatment of immune-mediated kidney diseases such as MN (40, 41).

### 1.3 Traditional Chinese medicine as a source of immunomodulatory compounds

Natural products have historically served as an important reservoir for drug discovery, particularly in the development of immunomodulatory and anti-inflammatory agents used in the treatment of complex immune-mediated diseases (42). A substantial proportion of modern pharmacological agents, including immunosuppressive drugs, antibiotics, and anticancer compounds, originate from natural sources or their derivatives, highlighting the therapeutic potential of bioactive molecules isolated from medicinal plants and fungi (43). In recent decades, increasing attention has been directed toward compounds derived from TCM, which contain diverse chemical classes such as flavonoids, alkaloids, terpenoids, polysaccharides, and nucleoside analogues with experimentally demonstrated immunological activity (44). Many TCM-derived compounds exhibit immunomodulatory properties by targeting key signaling pathways involved in inflammation, immune cell activation, and oxidative stress. Experimental studies have shown that several bioactive molecules isolated from medicinal plants regulate nuclear factor-κB (NF-κB), phosphoinositide 3-kinase/protein kinase B (PI3K/Akt), AMP-activated protein kinase (AMPK), and NOD-like receptor family pyrin domain containing 3 (NLRP3) inflammasome pathways, which play central roles in immune regulation and inflammatory responses (45, 46). These molecular targets are particularly relevant in immune-mediated kidney diseases because dysregulation of inflammatory signaling and innate immune activation contributes to the progression of glomerular injury and podocyte dysfunction (38, 47). In the context of membranous nephropathy, chronic immune activation, complement-mediated inflammation, and oxidative stress collectively contribute to podocyte damage and progressive proteinuria. Consequently, compounds capable of modulating inflammatory cytokine production, regulating immune cell function, and protecting podocyte integrity have attracted increasing interest as potential therapeutic candidates (16). A growing body of experimental evidence suggests that several well-characterized compounds derived from TCM—including flavonoids such as icariin, triterpenoid saponins such as astragaloside IV, iridoid glycosides such as catalpol, and nucleoside analogues such as cordycepin—exert immunoregulatory effects through defined molecular mechanisms (48, 49). Importantly, advances in modern pharmacology, metabolomics, and network pharmacology have enabled the identification of specific active constituents within traditional herbal medicines and the characterization of their molecular targets (50, 51). These approaches facilitate the transition from empirical herbal usage toward mechanism-based drug discovery by linking individual bioactive compounds with specific immunological pathways and disease processes (52). Such mechanistic insights are particularly valuable in autoimmune diseases, where therapeutic efficacy often depends on precise modulation of immune signaling networks rather than broad immunosuppression. Collectively, these developments highlight the growing relevance of TCM-derived bioactive compounds as a source of novel immunomodulatory agents. By integrating modern molecular pharmacology with traditional medicinal knowledge, it is possible to identify compounds with defined chemical structures and experimentally validated mechanisms that may provide new therapeutic opportunities for immune-mediated kidney diseases such as MN (39). In order to maintain mechanistic clarity and translational relevance, this review focuses exclusively on bioactive compounds with defined molecular structures and experimentally validated mechanisms of action affecting immune and inflammatory pathways.

### 1.4 Objective of the review

MN is increasingly recognized as a prototypical antibody-mediated autoimmune kidney disease in which dysregulated humoral immunity, complement activation, and podocyte injury interact to drive disease progression (8). Although advances in immunosuppressive therapy have improved disease management, the limitations of current treatments including incomplete remission, relapse, and treatment-related toxicity highlight the need for alternative therapeutic strategies that can modulate immune responses with greater specificity and reduced systemic adverse effects (22, 27). Natural products and bioactive molecules derived from traditional medicinal systems have emerged as promising sources of pharmacologically active compounds capable of targeting immune and inflammatory pathways relevant to autoimmune diseases (42). In particular, compounds isolated from TCM have attracted increasing scientific interest due to their diverse chemical structures and experimentally demonstrated biological activities, including regulation of inflammatory signaling, oxidative stress responses, and immune cell function (43). Advances in phytochemistry, molecular pharmacology, and systems biology have facilitated the identification of specific active constituents within complex herbal preparations and enabled the characterization of their molecular targets and mechanisms of action (52, 53). The purpose of the present review is therefore to systematically examine bioactive compounds derived from TCM that possess defined molecular structures and experimentally validated immunomodulatory mechanisms relevant to immune-mediated kidney diseases. Specifically, this review aims to (i) summarize the current understanding of the immunopathogenesis of MN, (ii) identify key bioactive compounds isolated from TCM that regulate immune and inflammatory signaling pathways, and (iii) discuss how these compounds may influence molecular mechanisms involved in MN pathogenesis, including immune cell activation, complement-mediated inflammation, and podocyte injury. By integrating insights from modern immunology, pharmacology, and natural product research, this review seeks to provide a mechanistic framework linking molecularly characterized TCM-derived compounds with immune regulatory pathways relevant to MN. Such an approach may facilitate the identification of novel therapeutic candidates and support the rational development of targeted immunomodulatory strategies for the treatment of autoimmune kidney diseases (39, 40).

## 2 Immunopathogenesis of membranous nephropathy

### 2.1 Autoantibody formation

**Figure 1:**
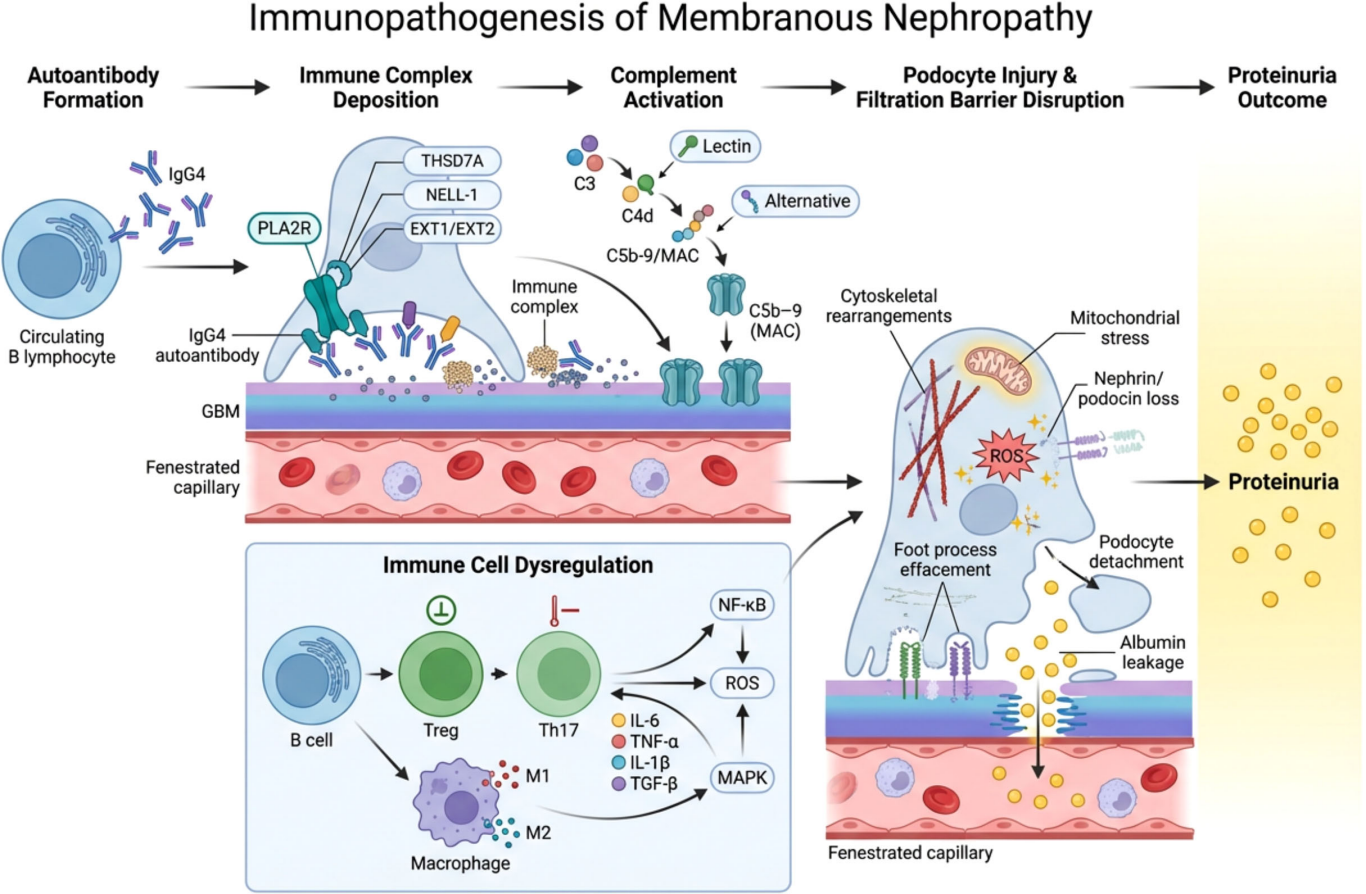
Immunopathogenesis of membranous nephropathy. The development of membranous nephropathy is initiated by the formation of autoantibodies produced by circulating B lymphocytes against podocyte antigens, most commonly the M-type phospholipase A2 receptor (PLA2R), and less frequently THSD7A, NELL-1, or EXT1/EXT2. Binding of these antibodies to podocyte antigens results in the formation of subepithelial immune complexes along the glomerular basement membrane (GBM). These immune complexes activate complement pathways, including the lectin and alternative pathways, leading to generation of the C5b-9 membrane attack complex (MAC). Complement-mediated injury induces cytoskeletal rearrangement, mitochondrial stress, and disruption of slit diaphragm proteins such as nephrin and podocin. Concurrent immune dysregulation involving B cells, T helper cell subsets, and macrophages promotes inflammatory signaling through pathways including NF-κB, MAPK, and reactive oxygen species (ROS) generation. These processes collectively result in podocyte injury, foot process effacement, and loss of filtration barrier integrity, ultimately leading to albumin leakage and clinical proteinuria.

Primary MN is widely recognized as an autoimmune glomerular disease driven by the formation of circulating autoantibodies directed against podocyte antigens located on the glomerular filtration barrier (4). Among the antigens identified to date, the M-type phospholipase A2 receptor (PLA2R) expressed on the surface of podocytes represents the principal target in the majority of patients with primary MN (7). The discovery of PLA2R as the dominant antigenic target has fundamentally advanced the understanding of MN pathogenesis and established the disease as a prototypical organ-specific autoimmune disorder (8). Anti-PLA2R autoantibodies are detected in approximately 70–80% of individuals with primary MN and correlate strongly with disease activity, proteinuria severity, and treatment response (54). These antibodies are predominantly of the IgG4 subclass, although other IgG subclasses such as IgG1 and IgG3 may also be present in certain stages of disease progression (55). The presence of circulating anti-PLA2R antibodies has therefore become an important serological biomarker for both diagnosis and monitoring of MN, allowing clinicians to assess immunological remission and predict the likelihood of disease relapse (56). Following their production by autoreactive B cells, anti-PLA2R antibodies bind to PLA2R molecules expressed on the surface of glomerular podocytes, leading to the formation of immune complexes directly at the site of antigen expression (5). This process, referred to as *in situ* immune complex formation, distinguishes MN from many other immune complex–mediated glomerular diseases in which circulating immune complexes deposit passively in renal tissues (3). The antigen–antibody interaction occurs along the subepithelial region of the glomerular basement membrane, resulting in characteristic granular immune deposits detectable by immunofluorescence microscopy (18). The accumulation of these immune complexes triggers a cascade of immunological events that ultimately disrupt the structural and functional integrity of the glomerular filtration barrier (57). Progressive deposition of immune complexes promotes activation of inflammatory pathways and initiates downstream complement activation, which contributes to podocyte injury and proteinuria (12). Importantly, experimental and clinical evidence indicates that the level of circulating anti-PLA2R antibodies closely reflects disease activity, and reductions in antibody titers frequently precede clinical remission following immunosuppressive therapy (58). Beyond PLA2R, additional podocyte antigens have been identified in a subset of MN patients, including thrombospondin type-1 domain–containing 7A (THSD7A) and several recently described antigens such as neural epidermal growth factor–like 1 protein (NELL-1) and exostosin 1/2 (EXT1/EXT2) (59). These findings further support the concept that MN represents a heterogeneous group of autoimmune podocytopathies characterized by antigen-specific autoantibody responses targeting components of the glomerular filtration barrier (60). The immunopathogenesis of MN involves autoantibody production against podocyte antigens, immune complex deposition along the glomerular basement membrane, complement activation, and subsequent podocyte injury leading to proteinuria (**Figure 1**). Several podocyte antigens have been identified as targets of pathogenic autoantibodies in membranous nephropathy, including PLA2R, THSD7A, NELL-1, and EXT1/EXT2, each associated with distinct immunological and clinical characteristics (**Table 1**).

**Table 1:** Major autoantigens and immune mechanisms in membranous nephropathy.

Antigen	Localization	Autoantibody class	Pathogenic mechanism	Refs
PLA2R (M-type phospholipase A2 receptor)	Podocyte membrane	IgG4 predominant	Formation of subepithelial immune complexes leading to complement activation and podocyte injury	(2, 61)
THSD7A (Thrombospondin type-1 domain containing 7A)	Podocyte surface	IgG4 predominant	Autoantibody-mediated immune complex deposition with complement activation	(62, 63)
NELL-1 (Neural epidermal growth factor-like 1)	Podocyte-associated antigen	IgG1 dominant	Immune complex deposition and activation of complement pathways	(64)
EXT1/EXT2	Glomerular basement membrane associated	Mixed subclasses	Associated with autoimmune-related membranous nephropathy	(65)

### 2.2 Complement activation and membrane attack complex formation (C5b-9)

Complement activation represents a central pathogenic mechanism in MN and plays a critical role in translating immune complex deposition into structural damage of the glomerular filtration barrier (12). Following the formation of subepithelial immune complexes between podocyte antigens and circulating autoantibodies, complement components are recruited and activated along the glomerular basement membrane, initiating a cascade of inflammatory and cytotoxic events (8). Immunofluorescence and proteomic analyses of kidney biopsy specimens consistently demonstrate deposition of complement components, including C3, C4d, and the terminal complement complex C5b–9, within the glomerular capillary walls of patients with MN (66). Although classical complement activation is commonly associated with immune complex diseases, accumulating evidence indicates that the lectin and alternative pathways play particularly important roles in MN pathogenesis (67). This is partly explained by the predominance of IgG4 autoantibodies in primary MN, which have limited ability to activate the classical complement pathway through C1q binding (8). Instead, mannose-binding lectin–dependent activation and amplification through the alternative pathway contribute significantly to complement cascade propagation and the generation of terminal complement complexes within glomerular tissues (12). The formation of the membrane attack complex (MAC), composed of complement components C5b–9, represents the final step of complement activation and is considered a key mediator of podocyte injury in MN (68). Unlike many complement-mediated diseases in which MAC induces direct cell lysis, sublytic deposition of C5b–9 on podocyte membranes triggers a variety of intracellular signaling events that disrupt cellular homeostasis (69). Experimental studies have demonstrated that MAC deposition induces cytoskeletal rearrangement, mitochondrial dysfunction, and activation of inflammatory signaling pathways within podocytes (70). Sublytic complement injury also stimulates the production of reactive oxygen species (ROS) and promotes activation of transcription factors such as nuclear factor-κB (NF-κB), leading to increased expression of pro-inflammatory cytokines and chemokines (71). These processes amplify local inflammatory responses within the glomerulus and contribute to progressive structural damage of podocytes and surrounding glomerular cells (72). Furthermore, complement-mediated signaling can alter the expression of key slit diaphragm proteins, including nephrin and podocin, thereby compromising the integrity of the filtration barrier and promoting the development of proteinuria (19). In addition to its direct cytotoxic effects on podocytes, complement activation also modulates immune cell recruitment and inflammatory responses within renal tissues. Complement fragments such as C3a and C5a function as potent anaphylatoxins that promote leukocyte chemotaxis, enhance inflammatory signaling, and amplify local immune responses (73). These mediators can further exacerbate glomerular inflammation and contribute to sustained immune-mediated injury in MN (74, 75). Collectively, these findings highlight complement activation—particularly the formation of the C5b–9 membrane attack complex—as a central pathogenic event linking autoantibody-mediated immune complex deposition to podocyte injury and glomerular dysfunction (76). Understanding the molecular pathways triggered by complement activation provides a critical framework for identifying therapeutic strategies capable of protecting podocytes and modulating inflammatory signaling in MN.

### 2.3 Immune cell dysregulation in membranous nephropathy

Beyond autoantibody production and complement activation, increasing evidence indicates that dysregulation of both innate and adaptive immune cells contributes significantly to the pathogenesis and progression of MN (13). The development of autoantibody responses against podocyte antigens is closely associated with abnormalities in B-cell activation, T-cell regulation, and macrophage-mediated inflammatory signaling within the renal microenvironment (8). These immune alterations promote chronic inflammation, amplify glomerular injury, and sustain autoimmune responses that ultimately damage the glomerular filtration barrier (77). B lymphocytes play a central role in MN pathogenesis because they are responsible for producing pathogenic autoantibodies targeting podocyte antigens such as PLA2R (78). Aberrant activation and differentiation of autoreactive B cells lead to the generation of plasma cells that secrete high-affinity IgG autoantibodies, which subsequently form immune complexes within the glomerular basement membrane (79). Clinical observations further support the importance of B cells in MN, as therapies that deplete B lymphocytes—such as rituximab—often result in a reduction of circulating anti-PLA2R antibodies and improvement in proteinuria (33). These findings underscore the critical contribution of B-cell–mediated humoral immunity to disease initiation and maintenance (80). T lymphocytes also play a crucial regulatory role in MN pathogenesis by modulating B-cell activation and shaping the inflammatory environment within renal tissues (55). Several studies have reported alterations in T-cell subsets in patients with MN, particularly an imbalance between regulatory T cells (Tregs) and pro-inflammatory T helper subsets such as Th17 cells (81). Tregs normally function to maintain immune tolerance and suppress autoreactive immune responses, whereas Th17 cells promote inflammatory cytokine production and tissue inflammation (82). A reduction in Treg activity combined with increased Th17 responses has been associated with enhanced autoimmune activity and persistent glomerular inflammation in MN patients (83). Macrophages represent another important component of the immune response in MN (84). These innate immune cells can adopt distinct functional phenotypes depending on microenvironmental signals, broadly categorized as pro-inflammatory M1 macrophages or anti-inflammatory and tissue-repair–associated M2 macrophages (85). In the context of MN, macrophage infiltration within glomerular and interstitial compartments contributes to inflammatory cytokine production and amplifies immune-mediated tissue injury (86). Activated macrophages release a variety of mediators—including tumor necrosis factor-α (TNF-α), interleukin-1β (IL-1β), and reactive oxygen species—which can exacerbate podocyte damage and disrupt the integrity of the glomerular filtration barrier (87). In addition to immune cell dysregulation, altered cytokine signaling plays an important role in sustaining inflammatory processes in MN (88). Elevated circulating and intrarenal levels of cytokines such as interleukin-6 (IL-6), TNF-α, and transforming growth factor-β (TGF-β) have been reported in patients with active disease and are associated with progressive glomerular injury and fibrosis (89). These inflammatory mediators can promote immune cell recruitment, enhance complement activation, and influence podocyte signaling pathways, thereby linking immune dysregulation with structural damage of the glomerulus (90). Taken together, these findings highlight that MN pathogenesis involves a complex network of immune cell interactions involving B cells, T lymphocytes, macrophages, and cytokine-mediated signaling pathways. Understanding these immune mechanisms is essential for identifying therapeutic strategies capable of restoring immune balance and preventing progressive glomerular injury in autoimmune kidney diseases.

### 2.4 Podocyte injury and glomerular filtration barrier disruption

Podocyte injury represents the final common pathological event in the development of proteinuria in MN and is a critical determinant of disease progression and renal dysfunction (17). Podocytes are highly specialized epithelial cells that line the outer surface of the glomerular capillaries and form an essential component of the glomerular filtration barrier through their interdigitating foot processes and slit diaphragm structures (19). Together with the glomerular basement membrane and fenestrated endothelial cells, podocytes maintain selective permeability of the filtration barrier, preventing the leakage of plasma proteins into the urinary space (91). In MN, immune complex deposition and complement activation trigger a series of cellular and molecular events that disrupt podocyte structure and function (92). Subepithelial immune deposits and the associated complement-mediated injury lead to cytoskeletal rearrangement within podocytes, resulting in effacement of foot processes and alterations in slit diaphragm integrity (18). These structural changes compromise the filtration barrier and represent a key mechanism underlying the development of massive proteinuria characteristic of MN (3). One of the earliest molecular events associated with podocyte injury is the alteration of key slit diaphragm proteins, including nephrin, podocin, and synaptopodin, which are essential for maintaining podocyte architecture and filtration barrier function (93). Complement-mediated signaling and inflammatory cytokines can downregulate the expression of these structural proteins, thereby weakening cell–cell junctions between adjacent podocyte foot processes (94). The resulting disruption of slit diaphragm integrity increases glomerular permeability and promotes leakage of albumin and other plasma proteins into the urine (94). In addition to structural damage, podocyte injury in MN is also associated with oxidative stress, mitochondrial dysfunction, and activation of intracellular inflammatory signaling pathways (95). Experimental studies have demonstrated that complement activation and immune complex deposition stimulate the generation of reactive oxygen species (ROS) within podocytes, leading to cellular stress responses and metabolic dysregulation (96). These processes activate downstream signaling pathways such as nuclear factor-κB (NF-κB) and mitogen-activated protein kinase (MAPK), which further amplify inflammatory responses and contribute to progressive glomerular injury (97). Persistent podocyte injury may ultimately lead to podocyte detachment from the glomerular basement membrane, an event that is considered irreversible because podocytes have limited capacity for regeneration (98). Loss of podocytes results in areas of denuded basement membrane that promote glomerulosclerosis and progressive deterioration of renal function (99). Consequently, the degree of podocyte depletion has been strongly associated with disease severity and long-term renal outcomes in patients with MN and other proteinuric kidney diseases (100). Collectively, these findings demonstrate that podocyte injury serves as the critical downstream consequence of autoimmune and complement-mediated processes in MN. The interplay between immune complex deposition, complement activation, inflammatory signaling, and oxidative stress ultimately converges on podocyte dysfunction, leading to disruption of the glomerular filtration barrier and the development of nephrotic syndrome (101). Understanding these mechanisms provides a mechanistic basis for therapeutic strategies aimed at protecting podocytes and preserving glomerular integrity in immune-mediated kidney diseases.

## 3 Conceptual bridge between TCM and modern immunology

### 3.1 Kidney essence (Jing) in traditional medicine

**Figure 2:**
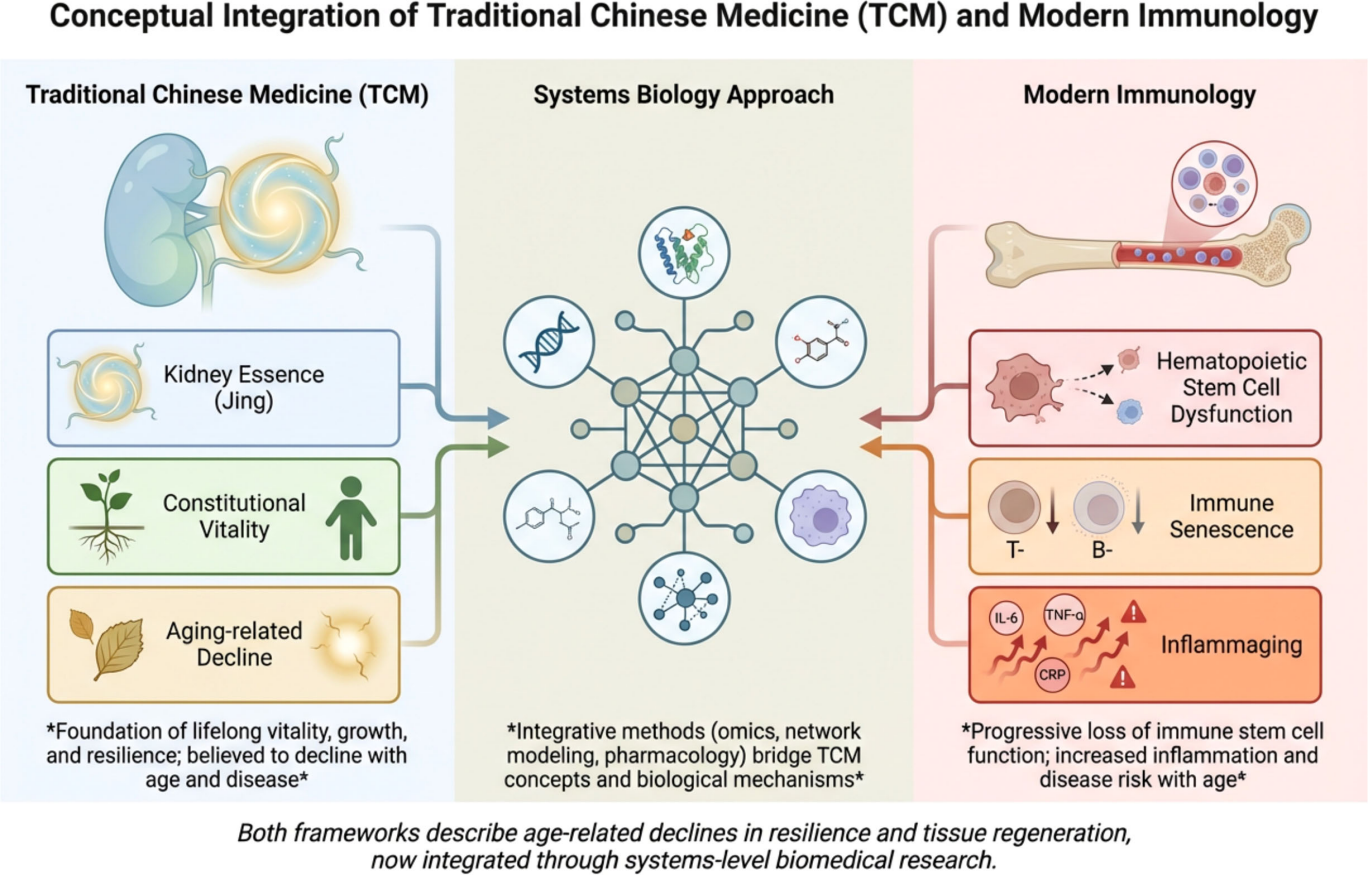
Conceptual integration of traditional Chinese medicine and modern immunology. This schematic illustrates the conceptual relationship between TCM theory and contemporary immunological understanding through a systems biology framework. In TCM, physiological resilience and long-term vitality are attributed to the concept of Kidney Essence (Jing), which is believed to support constitutional vitality and decline progressively with aging. From a modern biomedical perspective, age-associated functional decline may involve biological processes such as hematopoietic stem cell dysfunction, immune senescence, and chronic low-grade inflammation (inflammaging). Systems biology approaches—including multi-omics analysis, network modeling, and integrative pharmacology—provide methodological tools for bridging these conceptual frameworks by linking traditional medical theories with measurable molecular and cellular mechanisms. This integrative perspective highlights potential parallels between traditional descriptions of declining vitality and modern scientific understanding of aging-related immune and regenerative dysfunction.

TCM conceptualizes health and disease through a holistic framework in which physiological balance and constitutional resilience are maintained by fundamental life forces (102). Among these concepts, Kidney Essence (Jing) is considered one of the most fundamental substances governing growth, development, reproduction, and long-term vitality throughout the lifespan (103). In classical medical texts, Jing is described as the foundational biological reserve that supports organ function, tissue regeneration, and the body’s capacity to adapt to environmental stressors and pathological challenges (104). Jing is traditionally classified into two complementary components: prenatal essence (congenital Jing), which is inherited from parental sources and determines constitutional strength at birth, and postnatal essence, which is continuously replenished through nutrition, metabolism, and physiological activity during life (105). Together, these forms of essence are believed to sustain fundamental biological processes such as bone marrow production, growth and development, fertility, and systemic resilience against disease (106). Within the theoretical framework of TCM, the kidney is regarded as the organ system responsible for storing and regulating Jing, thereby influencing the body’s capacity for long-term physiological maintenance and recovery (107). An important feature of Jing is its close association with the processes of aging. Classical TCM literature describes aging as a gradual decline in kidney essence, leading to progressive deterioration of physiological functions and increased vulnerability to disease (108). Manifestations of Jing depletion are traditionally associated with symptoms such as skeletal weakness, fatigue, cognitive decline, infertility, and diminished resistance to illness (109). These descriptions reflect a conceptual framework in which aging is interpreted as a decline in the body’s constitutional reserves and regenerative capacity (110). Although Jing is not defined in biochemical terms, contemporary biomedical interpretations have attempted to draw parallels between this concept and physiological systems responsible for maintaining systemic homeostasis and regenerative potential (111). Some scholars have proposed that Jing may correspond functionally to biological processes such as stem cell maintenance, endocrine regulation, and immune resilience, which collectively sustain long-term organismal stability (112). From this perspective, the decline of Jing described in TCM may conceptually resemble the progressive deterioration of physiological systems observed during biological aging (113). Recent interdisciplinary discussions have therefore explored the possibility that traditional concepts such as Jing can be interpreted through modern scientific frameworks without assuming direct equivalence between the two paradigms (114). Such integrative perspectives aim to identify functional similarities between traditional medical theories and contemporary biological concepts, thereby facilitating cross-disciplinary dialogue between traditional medicine and modern biomedical research (115). TCM conceptualizes physiological resilience through the concept of Kidney Essence (Jing), which is believed to govern growth, vitality, and aging-related decline. From a modern biomedical perspective, these traditional concepts may be interpreted through systems-level biological mechanisms involving stem cell maintenance and immune regulation (**Figure 2**).

### 3.2 Hematopoietic stem cell dysfunction and immune senescence

In modern biomedical science, the maintenance of immune competence throughout life is largely dependent on the functional integrity of hematopoietic stem cells (HSCs), which reside primarily in the bone marrow and serve as the progenitors of all circulating immune cells (116). These multipotent stem cells continuously generate lymphoid and myeloid lineages, including T lymphocytes, B lymphocytes, natural killer (NK) cells, dendritic cells, and macrophages, thereby sustaining immune surveillance and host defense mechanisms (117). The capacity of HSCs to self-renew and differentiate is therefore essential for maintaining immune homeostasis and adaptive responses to pathogens and environmental stressors (118). During aging and chronic inflammatory conditions, however, the functional capacity of hematopoietic stem cells progressively declines (119). This process, commonly referred to as hematopoietic stem cell exhaustion, is characterized by reduced self-renewal potential, impaired differentiation capacity, and altered lineage commitment (120). Aging HSCs often exhibit a skewing toward myeloid lineage differentiation at the expense of lymphoid progenitors, resulting in decreased production of naïve T and B lymphocytes and reduced adaptive immune diversity (121). Such changes contribute to the phenomenon of immune senescence, a gradual deterioration of immune function that increases susceptibility to infections, malignancies, and autoimmune diseases (122). Immune senescence is associated with multiple molecular and cellular alterations, including telomere shortening, accumulation of DNA damage, mitochondrial dysfunction, and epigenetic remodeling within stem cell populations (123). These processes impair the regenerative capacity of hematopoietic stem cells and disrupt the delicate balance between immune activation and immune tolerance (124). Consequently, aging immune systems often display increased levels of systemic inflammation, a condition sometimes referred to as inflammaging, characterized by elevated circulating levels of pro-inflammatory cytokines such as interleukin-6 (IL-6), tumor necrosis factor-α (TNF-α), and C-reactive protein (125). The interplay between immune senescence and chronic inflammation has important implications for autoimmune kidney diseases such as MN (126). Persistent inflammatory signaling and immune dysregulation can promote autoreactive B-cell activation, aberrant antibody production, and sustained complement activation within renal tissues (13). Moreover, impaired immune regulation associated with aging may weaken mechanisms of immune tolerance, thereby increasing the likelihood of autoimmune responses directed against endogenous antigens, including podocyte-associated proteins (8). From an integrative perspective, the biological processes underlying HSC dysfunction and immune senescence may provide a potential conceptual parallel to the traditional notion of declining constitutional vitality described in TCM (127, 128). Although these frameworks arise from fundamentally different epistemological systems, both perspectives emphasize the progressive reduction of physiological resilience and regenerative capacity as key drivers of aging-related disease susceptibility (129). Recognizing such conceptual parallels may facilitate interdisciplinary dialogue and support the development of integrative research approaches aimed at understanding the complex interactions between immune aging, stem cell biology, and chronic disease (130).

### 3.3 Systems biology perspective: integrating traditional concepts with modern immunology

Advances in systems biology have provided powerful tools for understanding complex biological networks underlying immune regulation, aging, and chronic disease (131). Unlike traditional reductionist approaches that focus on single genes or molecular pathways, systems biology seeks to analyze biological processes as integrated networks of interacting components, including genes, proteins, metabolites, and signaling pathways (132). Such approaches are particularly valuable for studying complex diseases such as MN, in which immune dysregulation, complement activation, inflammatory signaling, and podocyte injury interact through highly interconnected molecular networks (133). High-throughput technologies—including genomics, transcriptomics, proteomics, and metabolomics—have significantly expanded the ability to investigate immune-mediated diseases at a systems level (134). These technologies enable comprehensive profiling of immune cell populations, inflammatory mediators, and metabolic pathways involved in disease pathogenesis. In the context of MN, multi-omics analyses have revealed complex alterations in immune signaling pathways, complement regulation, oxidative stress responses, and cellular metabolism that contribute to disease progression and renal injury (16). Such findings highlight the importance of integrative approaches capable of capturing the multifactorial nature of autoimmune kidney diseases. Systems biology has also played a central role in the emerging field of network pharmacology, which aims to understand how bioactive compounds interact with multiple molecular targets within biological networks (50). This paradigm is particularly relevant for natural products and traditional medicinal systems, where therapeutic effects often arise from simultaneous modulation of several signaling pathways rather than from single-target mechanisms (135). Network pharmacology integrates data from molecular docking, protein–protein interaction networks, pathway enrichment analyses, and systems-level modeling to predict how specific compounds influence disease-associated biological networks (136). In recent years, network-based approaches have been increasingly applied to investigate the pharmacological mechanisms of compounds derived from TCM (137). These studies have demonstrated that many bioactive molecules isolated from medicinal plants interact with multiple immune and inflammatory pathways, including NF-κB signaling, PI3K/Akt pathways, oxidative stress regulation, and inflammasome activation (52). Such multi-target pharmacological actions may be particularly advantageous in complex diseases such as MN, where immune dysregulation involves coordinated interactions among numerous molecular pathways (138). Importantly, systems biology approaches also facilitate the integration of traditional medical concepts with modern biomedical knowledge (139). By mapping the molecular targets of bioactive compounds onto disease-related signaling networks, researchers can explore how traditional therapeutic principles may correspond to measurable biological mechanisms (140). This integrative framework allows traditional medicinal knowledge to be examined through the lens of contemporary molecular science, thereby supporting the rational identification of pharmacologically active compounds with defined mechanisms of action (141). Collectively, systems biology and network pharmacology provide a conceptual and methodological bridge between traditional medical theories and modern immunological research. These approaches offer valuable strategies for identifying bioactive compounds capable of modulating complex immune networks involved in autoimmune diseases such as MN (142). Such integrative perspectives lay the foundation for the subsequent examination of molecularly characterized compounds derived from TCM and their potential immunomodulatory roles in the context of immune-mediated kidney disease.

## 4 Molecular immunomodulatory compounds derived from TCM

The increasing recognition of immune dysregulation as a central driver of MN has stimulated interest in identifying pharmacological agents capable of modulating inflammatory signaling pathways, complement activation, and immune cell responses (143). In recent years, bioactive molecules isolated from TCM have attracted considerable attention as potential immunomodulatory compounds with diverse chemical structures and experimentally validated biological activities (43, 144, 145). Advances in phytochemistry and molecular pharmacology have enabled the identification and characterization of numerous compounds derived from medicinal plants that exert regulatory effects on immune signaling pathways involved in inflammation and autoimmune disease (44). Several bioactive compounds derived from traditional Chinese medicinal plants have demonstrated immunomodulatory activity in experimental models, including icariin, astragaloside IV, catalpol, cordycepin, and Lycium barbarum polysaccharides (**Table 2**).

**Table 2:** Bioactive compounds derived from traditional Chinese medicine with immunomodulatory effects and their relevant to membranous nephropathy.

Compound	Chemical class	Source	Molecular formula	Major molecular targets	Immunological effects	Experimentally validated mechanisms	Refs
Icariin	Flavonoid glycoside	*Epimedium* species	C33H40O15	NF-κB, PI3K/Akt	Anti-inflammatory, cytokine suppression	Suppression of inflammatory cytokines, inhibition of NF-κB signaling, reduction of oxidative stress	(146–148)
Astragaloside IV	Triterpenoid saponin	*Astragalus membranaceus*	C41H68O14	PI3K/Akt, Treg modulation	Immune regulation, anti-inflammatory	Modulation of regulatory T cells, anti-inflammatory effects, protection against renal inflammation	(149)
Catalpol	Iridoid glycoside	*Rehmannia glutinosa*	C15H22O10	NF-κB, oxidative stress pathways	Anti-inflammatory, antioxidant	Inhibition of inflammatory cytokine production and enhancement of antioxidant defenses	(150, 151)
Cordycepin	Adenosine analogue	*Cordyceps militaris*	C10H13N5O3	AMPK, NLRP3 inflammasome	Anti-inflammatory, metabolic regulation	AMPK activation, suppression of inflammasome-mediated inflammation	(152, 153)
Lycium barbarum polysaccharides	Polysaccharide complex	*Lycium barbarum*	Heterogeneous	TLR4, NF-κB	Macrophage activation, immune modulation	Activation of macrophages and regulation of innate immune responses	(154, 155)

Unlike complex herbal formulations that contain multiple unidentified constituents, individual bioactive compounds isolated from medicinal plants can be studied using modern pharmacological and biochemical approaches (141). Such investigations allow the determination of chemical structures, molecular targets, and signaling pathways through which these compounds exert their biological effects (42). These mechanistic insights are essential for understanding how natural products influence immune responses and for evaluating their potential therapeutic relevance in immune-mediated diseases such as MN (156). A wide range of chemical classes derived from TCM have demonstrated immunomodulatory activity, including flavonoids, triterpenoid saponins, iridoid glycosides, polysaccharides, and nucleoside analogues (157). These compounds have been shown to regulate key signaling pathways involved in immune activation and inflammation, including nuclear factor-κB (NF-κB), phosphoinositide 3-kinase/protein kinase B (PI3K/Akt), mitogen-activated protein kinase (MAPK), and NOD-like receptor pyrin domain containing 3 (NLRP3) inflammasome pathways (158). Because these pathways play important roles in immune cell activation, cytokine production, oxidative stress responses, and inflammatory signaling, they represent important therapeutic targets in autoimmune kidney diseases (159). Experimental studies have demonstrated that several well-characterized TCM-derived compounds possess the ability to modulate immune responses, suppress inflammatory cytokine production, and protect renal cells from injury (160). These pharmacological effects are particularly relevant to MN, in which complement-mediated inflammation, immune cell activation, and oxidative stress contribute to podocyte damage and disruption of the glomerular filtration barrier (38). By targeting these mechanisms, bioactive natural compounds may offer potential therapeutic benefits either as adjunctive treatments or as lead molecules for the development of novel immunomodulatory drugs (161). In the following sections, this review focuses on selected TCM-derived compounds with clearly defined chemical structures and experimentally validated mechanisms of action. Emphasis is placed on compounds for which molecular pharmacological studies have demonstrated regulatory effects on immune signaling pathways relevant to autoimmune kidney disease and glomerular inflammation. The molecular mechanisms associated with these compounds have been investigated in a range of experimental systems, including *in vitro* immune cell assays, cultured podocyte models, and *in vivo* animal studies such as murine models of immune-mediated kidney injury. These studies have demonstrated that TCM-derived compounds can modulate inflammatory signaling pathways, regulate immune cell activity, and reduce oxidative stress responses, thereby providing mechanistic evidence supporting their potential therapeutic relevance in immune-mediated kidney diseases.

**Figure 3:**
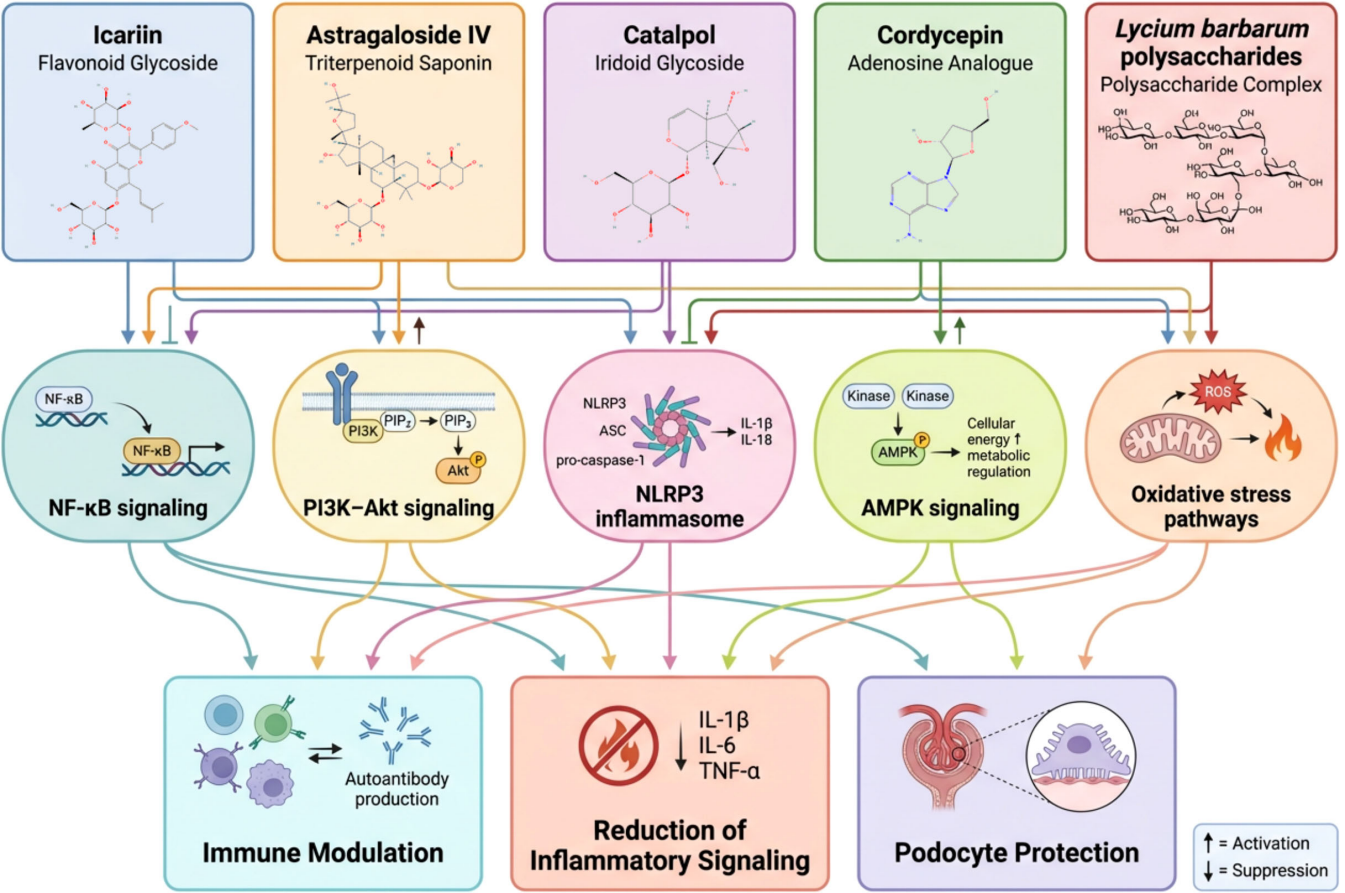
Molecular mechanisms of immunomodulatory compounds derived from traditional Chinese medicine. This schematic illustrates the principal signaling pathways targeted by selected bioactive compounds derived from traditional Chinese medicine. Icariin (a flavonoid glycoside), astragaloside IV (a triterpenoid saponin), catalpol (an iridoid glycoside), cordycepin (an adenosine analogue), and Lycium barbarum polysaccharides regulate multiple intracellular pathways involved in immune activation and inflammatory signaling. These pathways include NF-κB signaling, PI3K–Akt signaling, NLRP3 inflammasome activation, AMPK signaling, and oxidative stress–related pathways. Through modulation of these molecular networks, these compounds influence immune cell function, suppress the production of pro-inflammatory cytokines such as IL-1β, IL-6, and TNF-α, and enhance cellular stress responses. The combined effects of these mechanisms may contribute to immune modulation, attenuation of inflammatory signaling, and protection of podocytes from injury, processes that are relevant to the pathogenesis and potential treatment of immune-mediated kidney diseases such as membranous nephropathy. Upward arrows indicate pathway activation, whereas downward arrows indicate pathway inhibition.

Several bioactive compounds derived from traditional Chinese medicine—including icariin, astragaloside IV, catalpol, cordycepin, and Lycium barbarum polysaccharides—have been shown to modulate key molecular pathways involved in inflammation, immune regulation, and podocyte injury (**Figure 3**).

### 4.1 Icariin

Icariin is a prenylated flavonoid glycoside predominantly isolated from plants of the Epimedium genus, which have long been used in TCM for conditions associated with aging, bone degeneration, and immune dysfunction (162). Chemically, icariin belongs to the flavonol glycoside family and possesses the molecular formula C_33_H_40_O_15_. Its structure consists of a flavonoid backbone conjugated with sugar moieties and prenyl substituents that contribute to its biological activity and pharmacokinetic properties (163). Advances in phytochemistry and structural characterization have enabled detailed investigation of icariin’s molecular targets and pharmacological effects in various experimental disease models (146, 164). A growing body of experimental evidence indicates that icariin exerts significant immunomodulatory and anti-inflammatory effects through the regulation of multiple intracellular signaling pathways. One of the principal mechanisms involves inhibition of the nuclear factor-κB (NF-κB) signaling pathway, a key regulator of inflammatory gene expression and cytokine production (165). Activation of NF-κB plays a central role in many immune-mediated diseases by promoting transcription of pro-inflammatory mediators such as tumor necrosis factor-α (TNF-α), interleukin-6 (IL-6), and interleukin-1β (IL-1β) (166). Studies have demonstrated that icariin suppresses NF-κB activation by inhibiting phosphorylation of IκB kinase and preventing nuclear translocation of NF-κB transcription factors, thereby reducing inflammatory cytokine production in immune cells (167). In addition to its effects on inflammatory signaling, icariin has been shown to influence immune cell function and immune homeostasis. Experimental studies indicate that icariin can modulate macrophage activation, regulate T-cell responses, and enhance the balance between pro-inflammatory and anti-inflammatory cytokine production (168). These immunoregulatory properties may contribute to the compound’s protective effects in experimental models of inflammatory and autoimmune disorders (169). Icariin also exhibits antioxidant and cytoprotective activities that may be relevant to renal diseases characterized by oxidative stress and cellular injury (170). Research has demonstrated that icariin enhances the activity of endogenous antioxidant enzymes such as superoxide dismutase and catalase while reducing the accumulation of reactive oxygen species within cells (171). By attenuating oxidative stress and inflammatory signaling, icariin may help preserve cellular integrity and reduce tissue damage in conditions associated with chronic inflammation (172). Importantly, several experimental studies have reported protective effects of icariin in models of renal injury and glomerular disease (173). Icariin has been shown to attenuate renal inflammation, inhibit fibrotic signaling pathways, and reduce podocyte injury through modulation of signaling cascades such as NF-κB and PI3K/Akt pathways (174). These findings suggest that icariin may influence molecular mechanisms relevant to the pathogenesis of immune-mediated kidney diseases, including membranous nephropathy, where inflammatory signaling and oxidative stress contribute to podocyte dysfunction (175).

Collectively, these studies indicate that icariin represents a well-characterized natural compound with defined molecular structure and experimentally validated immunomodulatory mechanisms. Its ability to regulate inflammatory signaling pathways, modulate immune cell activity, and protect renal cells from injury highlights its potential relevance as a candidate molecule for further investigation in immune-mediated kidney diseases.

### 4.2 Astragaloside IV

Astragaloside IV is a cycloartane-type triterpenoid saponin isolated from the root of Astragalus membranaceus, a medicinal plant widely used in TCM for immune regulation and treatment of chronic inflammatory disorders (176). Structurally, astragaloside IV possesses the molecular formula C_41_H_68_O_14_ and consists of a cycloartane triterpenoid aglycone linked to multiple sugar moieties, a structural feature that contributes to its biological activity and pharmacological properties (177). Extensive phytochemical and pharmacological investigations have identified astragaloside IV as one of the principal active constituents responsible for the immunomodulatory effects of Astragalus species (178). A growing body of experimental evidence demonstrates that astragaloside IV exerts broad immunoregulatory effects by modulating several key signaling pathways involved in immune activation and inflammatory responses (178). One of the most extensively studied mechanisms involves regulation of the phosphoinositide 3-kinase/protein kinase B (PI3K/Akt) signaling pathway, which plays an essential role in immune cell survival, cytokine production, and inflammatory signaling (179). Activation of the PI3K/Akt pathway by astragaloside IV has been shown to regulate immune cell function and promote cellular survival under conditions of inflammatory stress (180). Astragaloside IV has also been reported to influence adaptive immune responses through modulation of regulatory T cells (Tregs) and inflammatory cytokine production (181). Experimental studies indicate that treatment with astragaloside IV can enhance the proportion and activity of Treg populations while simultaneously suppressing excessive inflammatory responses mediated by effector T cells (182). Such immunomodulatory effects may help restore immune tolerance and reduce autoimmune responses in chronic inflammatory conditions (169). In addition to its effects on immune cell regulation, astragaloside IV exhibits potent anti-inflammatory and antioxidant activities (178). Investigations in cellular and animal models have demonstrated that this compound suppresses the production of pro-inflammatory mediators—including tumor necrosis factor-α (TNF-α), interleukin-6 (IL-6), and interleukin-1β (IL-1β)—through inhibition of inflammatory signaling pathways such as nuclear factor-κB (NF-κB) and mitogen-activated protein kinase (MAPK) cascades (183). These anti-inflammatory actions may contribute to its protective effects in diseases characterized by chronic immune activation (184). Importantly, astragaloside IV has been widely studied in experimental models of renal disease, where it has demonstrated renoprotective properties through multiple mechanisms (185). These include attenuation of oxidative stress, inhibition of inflammatory signaling, reduction of renal fibrosis, and protection of podocytes from injury (186). Experimental studies have shown that astragaloside IV can stabilize podocyte structure, reduce inflammatory cytokine production in renal tissues, and improve renal function in animal models of glomerular injury (187). Because immune-mediated inflammation and podocyte damage represent central pathogenic events in membranous nephropathy, these findings suggest that astragaloside IV may have therapeutic relevance in immune-mediated kidney disorders (101). Collectively, astragaloside IV represents a well-characterized natural compound with defined chemical structure and experimentally validated immunomodulatory mechanisms. Its ability to regulate immune signaling pathways, modulate T-cell responses, and protect renal cells from inflammatory injury highlights its potential as a candidate molecule for further investigation in the context of autoimmune kidney diseases.

### 4.3 Catalpol

Catalpol is an iridoid glycoside predominantly isolated from the roots of Rehmannia glutinosa, a medicinal plant widely used in TCM for the treatment of metabolic, inflammatory, and aging-related disorders (188). Structurally, catalpol possesses the molecular formula C_15_H_22_O_10_ and belongs to the class of monoterpenoid iridoid glycosides characterized by a cyclopentanoid monoterpene core linked to a glucose moiety (189). Extensive phytochemical investigations have identified catalpol as one of the principal bioactive components responsible for the pharmacological effects attributed to Rehmannia glutinosa in traditional medicine (150, 190). Accumulating experimental evidence indicates that catalpol exerts significant anti-inflammatory and immunomodulatory effects through regulation of multiple intracellular signaling pathways (191, 192). One of the key mechanisms involves inhibition of inflammatory signaling mediated by nuclear factor-κB (NF-κB), a transcription factor that regulates the expression of numerous pro-inflammatory cytokines and chemokines (45). Studies have demonstrated that catalpol suppresses activation of the NF-κB pathway by inhibiting phosphorylation and degradation of inhibitor κB (IκB), thereby reducing nuclear translocation of NF-κB and attenuating transcription of inflammatory mediators such as tumor necrosis factor-α (TNF-α), interleukin-6 (IL-6), and interleukin-1β (IL-1β) (193). In addition to its anti-inflammatory actions, catalpol has been shown to regulate oxidative stress responses and mitochondrial function (194). Experimental studies indicate that catalpol enhances cellular antioxidant defenses by increasing the activity of endogenous antioxidant enzymes and reducing the accumulation of reactive oxygen species within cells (195). These antioxidant effects are partly mediated through activation of signaling pathways involved in cellular stress responses and mitochondrial homeostasis, thereby protecting cells from oxidative damage and metabolic dysfunction (196). Catalpol has also been reported to exert protective effects in experimental models of kidney injury and inflammatory renal disease. Research has shown that catalpol can attenuate renal inflammation, reduce oxidative stress, and inhibit fibrotic signaling pathways in models of chronic kidney disease and glomerular injury (197). In particular, catalpol has been demonstrated to protect renal cells by suppressing inflammatory cytokine production and reducing oxidative damage within renal tissues (198). These mechanisms may contribute to preservation of glomerular structure and function under inflammatory conditions. Furthermore, emerging evidence suggests that catalpol may influence immune regulation by modulating cytokine production and inflammatory signaling in immune cells (199). By suppressing excessive inflammatory responses while enhancing antioxidant defense mechanisms, catalpol may help restore immune balance in pathological conditions characterized by chronic inflammation and immune dysregulation (194). Such properties may be particularly relevant in autoimmune kidney diseases, where inflammatory signaling and oxidative stress contribute to podocyte injury and glomerular dysfunction. Collectively, these findings demonstrate that catalpol represents a well-characterized natural compound with defined molecular structure and experimentally validated anti-inflammatory and cytoprotective mechanisms. Through its ability to regulate inflammatory signaling pathways, modulate oxidative stress responses, and protect renal tissues from injury, catalpol may represent a promising candidate for further investigation in immune-mediated renal diseases.

### 4.4 Cordycepin

Cordycepin (3′-deoxyadenosine) is a nucleoside analogue originally isolated from the medicinal fungus Cordyceps militaris and related species that have been widely used in TCM for centuries in the treatment of fatigue, respiratory disorders, and immune-related diseases (200). Structurally, cordycepin is closely related to adenosine but lacks a hydroxyl group at the 3′ position of the ribose moiety, a modification that significantly influences its biological activity and interactions with cellular metabolic pathways (201). Owing to this structural similarity to endogenous nucleosides, cordycepin can interact with multiple molecular targets involved in cellular metabolism, immune signaling, and inflammatory regulation (202). A substantial body of experimental evidence demonstrates that cordycepin possesses potent anti-inflammatory and immunomodulatory properties (203). One of the most extensively studied mechanisms involves activation of the AMP-activated protein kinase (AMPK) signaling pathway, a central regulator of cellular energy homeostasis and metabolic stress responses (204). Activation of AMPK by cordycepin has been shown to inhibit inflammatory signaling cascades and suppress the production of pro-inflammatory cytokines in immune cells (205). Because AMPK signaling also influences oxidative stress responses and mitochondrial function, cordycepin may exert protective effects in inflammatory and metabolic disorders. Cordycepin has also been reported to inhibit activation of the NOD-like receptor pyrin domain containing 3 (NLRP3) inflammasome, an intracellular multiprotein complex that plays a key role in innate immune responses and inflammatory cytokine production (206). Activation of the NLRP3 inflammasome leads to cleavage of pro–interleukin-1β and pro–interleukin-18, resulting in the release of mature inflammatory cytokines that amplify immune responses and tissue injury (207). Experimental studies indicate that cordycepin can suppress NLRP3 inflammasome activation by modulating upstream signaling pathways, thereby reducing inflammatory cytokine release and attenuating inflammatory responses (208). In addition to these mechanisms, cordycepin has been shown to influence multiple cellular processes associated with immune regulation and tissue protection. These include inhibition of nuclear factor-κB (NF-κB) signaling, reduction of oxidative stress, and modulation of macrophage activation and cytokine production (209). Through these actions, cordycepin can attenuate inflammatory signaling and promote cellular homeostasis under conditions of immune activation and oxidative stress (203). Cordycepin has also demonstrated protective effects in experimental models of renal injury and inflammatory kidney disease. Studies have reported that treatment with cordycepin can reduce renal inflammation, inhibit fibrotic signaling pathways, and improve renal function in models of kidney damage (210). These effects are associated with suppression of inflammatory cytokine production, inhibition of inflammasome activation, and reduction of oxidative stress within renal tissues (211). Given that inflammatory signaling, complement activation, and oxidative stress contribute to podocyte injury in membranous nephropathy, these findings suggest that cordycepin may have potential relevance in immune-mediated renal diseases (212). Overall, cordycepin represents a well-characterized bioactive compound with a defined molecular structure and multiple experimentally validated immunomodulatory mechanisms. Its ability to regulate inflammatory signaling pathways, inhibit inflammasome activation, and modulate immune cell responses highlights its potential as a promising candidate for further investigation in the context of autoimmune and inflammatory kidney diseases.

### 4.5 Lycium barbarum polysaccharides

Lycium barbarum polysaccharides (LBPs) are a group of bioactive macromolecules isolated from the fruit of Lycium barbarum (commonly known as goji berry), a medicinal plant widely used in TCM for promoting longevity, immune resilience, and metabolic health (213). Chemically, LBPs consist of heterogeneous polysaccharide–protein complexes composed primarily of monosaccharides such as arabinose, galactose, glucose, rhamnose, mannose, and xylose arranged in branched glycosidic structures (214). Structural analyses using chromatographic and spectroscopic techniques have revealed that these polysaccharides possess complex three-dimensional conformations that contribute to their immunological activity and biological functions (215). A substantial body of experimental evidence indicates that LBPs exhibit pronounced immunomodulatory properties, particularly through their ability to regulate innate immune responses (216). One of the principal mechanisms involves activation of macrophages through pattern recognition receptors such as Toll-like receptor 4 (TLR4), which initiates downstream signaling cascades associated with innate immune activation (217). Engagement of TLR4 by LBP components can stimulate intracellular signaling pathways including nuclear factor-κB (NF-κB) and mitogen-activated protein kinase (MAPK), leading to increased production of cytokines and immune mediators involved in host defense (218). In addition to macrophage activation, LBPs have been shown to influence the function of other immune cell populations, including dendritic cells, T lymphocytes, and natural killer (NK) cells (219). Experimental studies indicate that LBP treatment enhances dendritic cell maturation and antigen-presenting capacity, thereby promoting adaptive immune responses (220). Furthermore, LBPs have been reported to stimulate T-cell proliferation and increase the production of cytokines such as interleukin-2 (IL-2) and interferon-γ (IFN-γ), which are essential for effective cellular immune responses (221). LBPs also possess notable antioxidant and cytoprotective properties that contribute to their immunological effects. Investigations have demonstrated that these polysaccharides can enhance cellular antioxidant defenses by increasing the activity of enzymes such as superoxide dismutase and glutathione peroxidase while reducing the accumulation of reactive oxygen species within tissues (222). Through modulation of oxidative stress pathways, LBPs may help protect immune cells and other tissues from damage associated with chronic inflammation and metabolic stress (223). In the context of aging and immune senescence, LBPs have attracted considerable attention due to their potential to enhance immune function and restore immune balance in aging organisms. Studies in experimental models have shown that LBP administration can improve immune responses, enhance lymphocyte proliferation, and increase macrophage phagocytic activity in aged animals (218). These effects suggest that LBPs may help counteract age-related immune decline and improve immune resilience under conditions of physiological stress (224). Although direct studies in MN remain limited, the ability of LBPs to regulate innate and adaptive immune responses, reduce oxidative stress, and modulate inflammatory signaling pathways suggests potential relevance for immune-mediated kidney diseases. By influencing immune cell activation and inflammatory cytokine production, LBPs may contribute to restoring immune balance in conditions characterized by chronic inflammation and immune dysregulation. Overall, Lycium barbarum polysaccharides represent a class of bioactive natural compounds with well-documented immunomodulatory effects and defined structural characteristics. Their ability to regulate immune signaling pathways, enhance immune cell function, and modulate oxidative stress responses highlights their potential relevance in the broader context of immune regulation and inflammatory disease.

## 5 Molecular pathways targeted by TCM-derived compounds

The immunomodulatory effects of bioactive compounds derived from TCM are mediated through the regulation of multiple intracellular signaling pathways that govern immune cell activation, inflammatory responses, and cellular stress adaptation. Unlike many conventional pharmacological agents that act on single molecular targets, natural compounds frequently influence several interconnected signaling cascades simultaneously, thereby modulating complex biological networks involved in immune regulation (50). These bioactive compounds exert their biological effects through modulation of multiple intracellular signaling pathways involved in inflammation, immune regulation, and cellular stress responses, including NF-κB, PI3K–Akt, NLRP3 inflammasome, AMPK signaling, and oxidative stress pathways (**Table 3**). Such multi-target mechanisms may be particularly advantageous in immune-mediated diseases such as MN, where disease progression results from the interaction of numerous inflammatory, immunological, and cellular injury pathways. Experimental studies investigating compounds such as icariin, astragaloside IV, catalpol, cordycepin, and Lycium barbarum polysaccharides have demonstrated that these molecules regulate key signaling pathways associated with inflammation, immune cell function, oxidative stress, and cellular metabolism. The principal pathways affected include nuclear factor-κB (NF-κB) signaling, phosphoinositide 3-kinase/protein kinase B (PI3K/Akt) signaling, NOD-like receptor pyrin domain containing 3 (NLRP3) inflammasome activation, AMP-activated protein kinase (AMPK) signaling, and oxidative stress–related pathways (52). These pathways are known to play central roles in autoimmune responses, inflammatory signaling, and podocyte injury in MN.

**Table 3:** Molecular pathways targeted by TCM-derived compounds relevant to MN pathogenesis.

Pathway	Biological role in MN	Representative compounds	Therapeutic implication	Refs
NF-κB signaling	Regulation of inflammatory cytokine expression	Icariin, Catalpol	Reduction of inflammatory responses	(225)
PI3K/Akt signaling	Cell survival and immune regulation	Astragaloside IV	Protection against podocyte injury	(180)
NLRP3 inflammasome	Activation of IL-1β and inflammatory responses	Cordycepin	Suppression of inflammasome activation	(152)
AMPK signaling	Cellular metabolism and anti-inflammatory signaling	Cordycepin	Metabolic regulation and inflammation control	(226)
Oxidative stress pathways	ROS generation and podocyte damage	Icariin, Catalpol, LBP	Antioxidant protection	(227)

### 5.1 NF-κB signaling pathway

The NF-κB signaling pathway is one of the most important regulators of inflammatory gene expression and immune cell activation. Activation of NF-κB leads to transcription of numerous pro-inflammatory cytokines, chemokines, and adhesion molecules that contribute to immune-mediated tissue injury (228). In renal diseases, NF-κB activation has been implicated in the pathogenesis of glomerular inflammation, podocyte injury, and progressive renal fibrosis (90). Several TCM-derived compounds discussed in this review have been shown to suppress NF-κB signaling. For example, icariin and catalpol inhibit phosphorylation of inhibitor κB (IκB) and prevent nuclear translocation of NF-κB transcription factors, thereby reducing expression of inflammatory cytokines such as TNF-α, IL-6, and IL-1β (229). Similarly, cordycepin has been reported to attenuate inflammatory signaling through inhibition of NF-κB activation in immune cells and inflammatory tissues (230). Through these mechanisms, natural compounds may reduce inflammatory responses that contribute to immune-mediated glomerular injury.

### 5.2 PI3K/Akt signaling pathway

The phosphoinositide 3-kinase/protein kinase B (PI3K/Akt) signaling pathway plays a fundamental role in regulating cell survival, metabolism, and immune responses. Activation of this pathway influences immune cell proliferation, cytokine production, and inflammatory signaling (231). Dysregulation of PI3K/Akt signaling has been implicated in various inflammatory and autoimmune diseases, including kidney disorders characterized by immune-mediated tissue injury. Astragaloside IV has been widely reported to regulate PI3K/Akt signaling, contributing to its anti-inflammatory and cytoprotective effects (232). Activation of the PI3K/Akt pathway by astragaloside IV can promote cell survival, reduce inflammatory cytokine production, and enhance cellular resistance to oxidative stress. These effects may help protect renal cells from injury in inflammatory environments associated with autoimmune kidney diseases.

### 5.3 NLRP3 inflammasome

The NLRP3 inflammasome is a multiprotein complex that plays a critical role in innate immune responses by regulating activation of inflammatory cytokines such as interleukin-1β and interleukin-18 (206). Activation of the NLRP3 inflammasome promotes inflammatory signaling and has been implicated in a variety of inflammatory and autoimmune diseases, including kidney injury and glomerular inflammation (233). Experimental studies have demonstrated that cordycepin can suppress activation of the NLRP3 inflammasome, thereby reducing production of inflammatory cytokines and attenuating inflammatory responses (208). By inhibiting inflammasome activation, cordycepin may help limit inflammatory signaling pathways that contribute to renal tissue injury.

### 5.4 AMPK signaling pathway

AMP-activated protein kinase (AMPK) is a key regulator of cellular energy homeostasis and metabolic stress responses. Activation of AMPK has been shown to exert anti-inflammatory effects, regulate cellular metabolism, and protect tissues from oxidative damage (234). Increasing evidence indicates that AMPK signaling also plays an important role in regulating immune cell activation and inflammatory responses. Cordycepin has been reported to activate AMPK signaling, which contributes to suppression of inflammatory cytokine production and regulation of immune responses (235). Through activation of AMPK-dependent pathways, cordycepin may enhance cellular stress resistance and reduce inflammatory signaling in immune-mediated diseases (236).

### 5.5 Oxidative stress and antioxidant pathways

Oxidative stress is an important contributor to inflammation and cellular injury in many chronic diseases, including kidney disorders. Excessive production of reactive oxygen species can damage cellular components, disrupt mitochondrial function, and amplify inflammatory signaling pathways (237). In MN, oxidative stress has been associated with podocyte injury and progressive deterioration of glomerular structure and function. Several natural compounds derived from TCM exhibit antioxidant properties that help reduce oxidative stress and protect cells from inflammatory injury. For example, icariin, catalpol, and Lycium barbarum polysaccharides have been shown to enhance the activity of endogenous antioxidant enzymes and reduce reactive oxygen species accumulation in experimental models (238). By modulating oxidative stress pathways, these compounds may contribute to preservation of cellular integrity and reduction of inflammatory damage (239). Collectively, these findings demonstrate that TCM-derived compounds regulate multiple molecular pathways involved in immune activation, inflammatory signaling, and cellular stress responses. Because MN pathogenesis involves complex interactions among immune cells, complement activation, and podocyte injury, compounds capable of simultaneously modulating several signaling pathways may offer potential advantages for the development of novel therapeutic strategies targeting immune-mediated kidney disease.

## 6 Translational potential of TCM-derived compounds in membranous nephropathy

The pathogenesis of MN involves a complex interplay of immune dysregulation, complement activation, inflammatory signaling, and podocyte injury. Although current therapeutic strategies including immunosuppressive agents such as rituximab, cyclophosphamide, and calcineurin inhibitors have improved clinical outcomes, these treatments remain associated with significant adverse effects and variable efficacy among patients (35). Consequently, there is growing interest in identifying complementary pharmacological strategies capable of modulating disease-relevant molecular pathways while minimizing systemic toxicity. Bioactive compounds derived from TCM may offer potential translational relevance in this context. As discussed in previous sections, several well-characterized natural compounds—including icariin, astragaloside IV, catalpol, cordycepin, and Lycium barbarum polysaccharides—have demonstrated experimentally validated immunomodulatory and cytoprotective properties. These compounds regulate key signaling pathways involved in inflammatory responses, immune cell activation, oxidative stress regulation, and cellular metabolism. Because these pathways are directly implicated in MN pathogenesis, modulation of these molecular mechanisms may contribute to protective effects within the renal microenvironment.

### 6.1 Targeting podocyte injury

Podocyte injury represents a central pathological event in MN and serves as the primary driver of proteinuria and progressive glomerular damage. Therapeutic strategies aimed at protecting podocyte structure and function are therefore considered critical for preventing disease progression (240). Experimental studies have shown that several natural compounds can protect podocytes by attenuating inflammatory signaling, reducing oxidative stress, and stabilizing cytoskeletal structures within these specialized cells. For example, astragaloside IV and icariin have been reported to reduce inflammatory cytokine production and improve cellular resistance to oxidative stress in experimental models of renal injury (186). These effects may help preserve podocyte integrity by preventing cytoskeletal disruption and maintaining slit diaphragm protein expression. Similarly, catalpol and cordycepin have demonstrated protective effects against mitochondrial dysfunction and inflammatory signaling pathways that contribute to podocyte damage (241). Through these mechanisms, natural compounds may help maintain the structural integrity of the glomerular filtration barrier.

### 6.2 Regulation of immune tolerance and inflammatory signaling

Restoration of immune balance represents another important therapeutic objective in MN. The disease is characterized by dysregulated immune responses, including autoreactive B-cell activation, altered T-cell regulation, and complement-mediated inflammatory injury (242). Compounds capable of modulating immune signaling pathways may therefore contribute to restoring immune homeostasis (243). Several TCM-derived compounds have demonstrated the ability to regulate immune cell function and cytokine production. Astragaloside IV has been reported to enhance regulatory T-cell activity while suppressing excessive inflammatory responses, suggesting potential roles in promoting immune tolerance (182). Likewise, Lycium barbarum polysaccharides have been shown to modulate macrophage activation and influence cytokine production through Toll-like receptor signaling pathways (244). By influencing both innate and adaptive immune responses, these compounds may contribute to normalization of immune regulation in inflammatory conditions.

### 6.3 Potential combination with existing therapies

Another potential translational application of natural immunomodulatory compounds involves their use as adjunctive agents alongside conventional therapies. Current MN treatments often rely on broad immunosuppression, which can increase the risk of infection and other systemic complications (245). Compounds that selectively regulate inflammatory signaling pathways or protect renal cells from injury may complement existing therapies by reducing inflammatory damage while minimizing systemic immune suppression (159). In experimental settings, several natural compounds have demonstrated synergistic or additive effects when combined with conventional pharmacological agents targeting inflammatory pathways (246). Such combinational approaches may allow lower doses of immunosuppressive drugs to be used while maintaining therapeutic efficacy. However, further pharmacological and clinical investigations are required to evaluate the safety, pharmacokinetics, and therapeutic potential of these compounds in human patients with MN (247). Overall, the growing body of experimental evidence suggests that bioactive compounds derived from traditional medicinal plants may have potential relevance for modulating molecular pathways involved in MN pathogenesis. While most available data currently derive from preclinical studies, continued investigation of these compounds may contribute to the development of novel therapeutic strategies targeting immune-mediated kidney diseases.

## 7 Systems biology and precision medicine

The growing integration of systems biology and precision medicine has transformed the investigation of complex diseases by enabling the comprehensive analysis of molecular networks underlying disease development and progression. In contrast to traditional approaches that focus on isolated molecular targets, systems-level methodologies allow researchers to examine interactions among genes, proteins, metabolites, and signaling pathways within the broader biological context of disease (248). Such integrative strategies are particularly relevant for immune-mediated disorders such as MN, in which immune dysregulation, complement activation, inflammatory signaling, and podocyte injury arise from highly interconnected biological networks. Recent advances in systems biology including multi-omics technologies, biomarker discovery, machine learning–based disease classification, and network pharmacology have created new opportunities for precision medicine approaches in MN (**Table 4**).

**Table 4:** Emerging precision medicine approaches in membranous nephropathy.

Approach	Technology	Clinical application	Refs
Multi-omics profiling	Genomics, transcriptomics, proteomics	Identification of disease mechanisms	(249)
Biomarker-guided therapy	Anti-PLA2R monitoring	Patient stratification and treatment monitoring	(250)
AI-assisted classification	Machine learning	Identification of disease subtypes	(251)
Network pharmacology	Computational drug discovery	Identification of multi-target compounds	(252)

Precision medicine approaches aim to tailor therapeutic strategies according to individual molecular and clinical characteristics of patients. In the context of autoimmune kidney diseases, the identification of specific biomarkers—such as circulating autoantibodies, complement activation profiles, and immune signaling signatures—has already begun to influence diagnostic and therapeutic decision-making (55). For example, detection of circulating anti-PLA2R antibodies has become an important diagnostic and prognostic biomarker in primary MN, reflecting underlying autoimmune activity and disease progression (7). Integrating molecular biomarkers with clinical parameters may therefore facilitate improved disease stratification and personalized therapeutic interventions. Systems biology approaches can also support the identification of molecular targets influenced by bioactive natural compounds. Because many natural products exert their biological effects through modulation of multiple molecular pathways, integrative network analyses provide valuable tools for understanding how such compounds influence disease-associated signaling networks (135). In this context, systems pharmacology and network-based analyses have increasingly been applied to explore the interactions between bioactive compounds derived from traditional medicinal plants and molecular pathways involved in inflammatory and autoimmune diseases.

**Figure 4:**
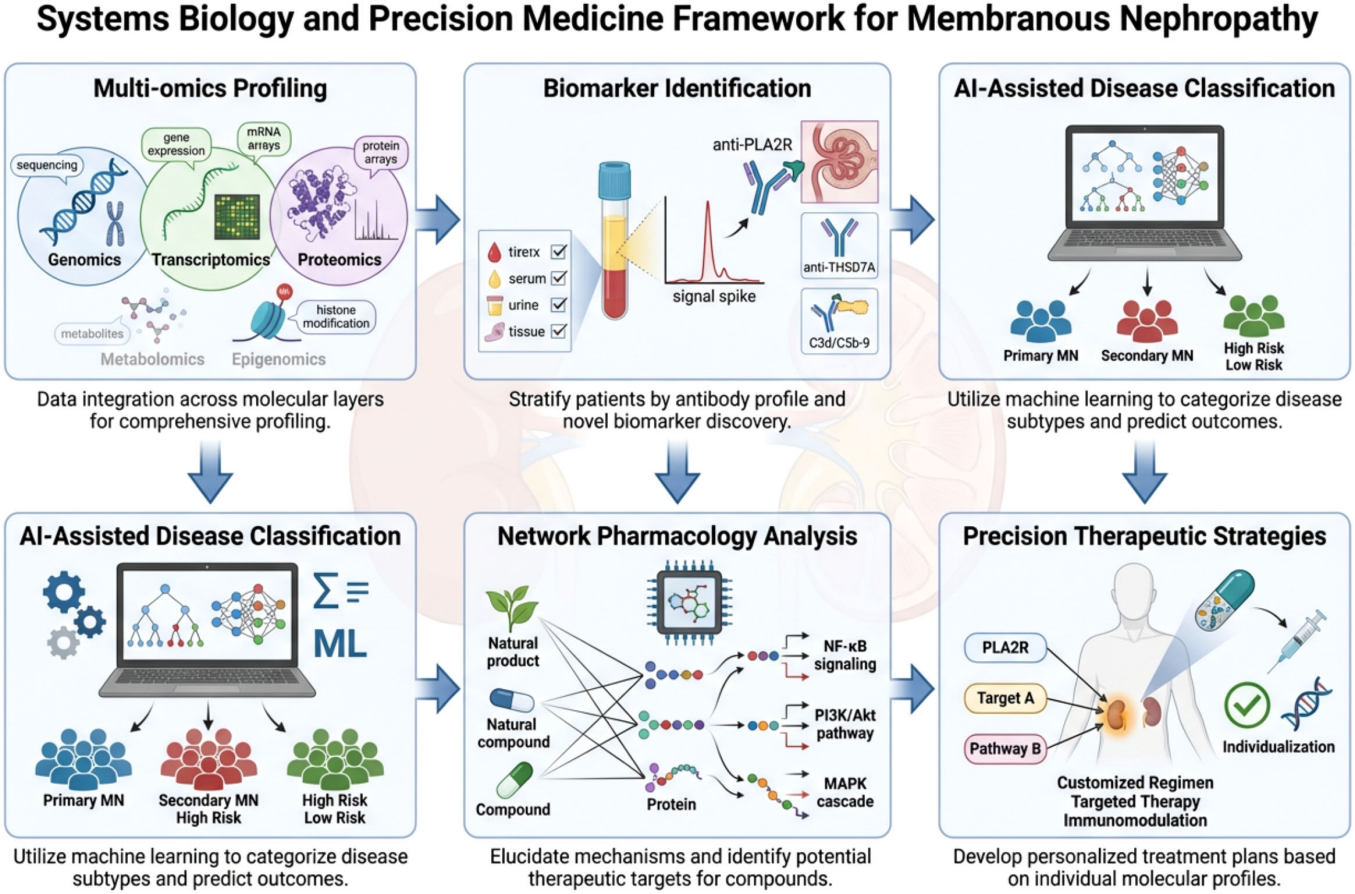
Systems biology and precision medicine framework for membranous nephropathy. This schematic illustrates an integrative framework for applying systems biology and precision medicine approaches to membranous nephropathy (MN). Multi-omics profiling—including genomics, transcriptomics, proteomics, metabolomics, and epigenomics—enables comprehensive molecular characterization of disease processes. Biomarker identification, including antibodies such as anti-PLA2R and anti-THSD7A as well as complement components, supports patient stratification and disease monitoring. Artificial intelligence and machine learning algorithms can integrate multi-layered datasets to classify disease subtypes, distinguish primary and secondary MN, and predict clinical outcomes. Network pharmacology approaches further facilitate the identification of molecular targets and signaling pathways associated with bioactive compounds and therapeutic interventions. Together, these integrative strategies enable the development of precision therapeutic approaches, including targeted immunomodulation and individualized treatment regimens based on patient-specific molecular profiles.

Recent advances in multi-omics technologies, biomarker discovery, and computational modeling have enabled the development of systems biology frameworks that integrate molecular data with clinical phenotypes to support precision medicine approaches in MN (**Figure 4**).

### 7.1 Multi-omics approaches

Multi-omics technologies represent one of the most powerful methodological advances in systems biology. These approaches integrate data from multiple molecular layers—including genomics, transcriptomics, proteomics, metabolomics, and epigenomics—to generate a comprehensive view of biological processes and disease mechanisms (253). By combining these complementary datasets, researchers can identify complex molecular interactions and regulatory networks that may not be detectable using single-layer analyses. In the study of autoimmune kidney diseases, multi-omics analyses have revealed important insights into immune cell regulation, complement pathway activation, and metabolic alterations associated with glomerular injury. Transcriptomic profiling of kidney biopsy samples has identified gene expression signatures associated with inflammatory signaling, complement activation, and immune cell infiltration in patients with MN (254). Similarly, proteomic studies have detected alterations in complement proteins, immunoglobulins, and inflammatory mediators within glomerular tissues and urine samples, providing potential biomarkers for disease activity and progression (255). Metabolomic investigations have further highlighted metabolic disturbances associated with immune activation and oxidative stress in kidney disease. Altered metabolic pathways—including those related to lipid metabolism, mitochondrial function, and energy regulation—have been implicated in the progression of glomerular injury and renal dysfunction (256). Integrating metabolomic data with transcriptomic and proteomic information allows researchers to construct systems-level models of disease mechanisms and identify novel therapeutic targets. The application of multi-omics approaches may also facilitate the investigation of natural compounds with immunomodulatory properties. By analyzing molecular changes induced by specific compounds across multiple biological layers, researchers can identify signaling networks and metabolic pathways influenced by these molecules (257). Such integrative analyses may help clarify how bioactive compounds derived from traditional medicinal plants influence immune regulation and inflammatory processes relevant to autoimmune diseases. Overall, the integration of multi-omics technologies within systems biology frameworks provides a powerful strategy for advancing precision medicine in immune-mediated kidney diseases. These approaches enable deeper understanding of disease heterogeneity, identification of molecular biomarkers, and discovery of novel therapeutic targets that may support the development of personalized treatment strategies in conditions such as membranous nephropathy (258).

### 7.2 Biomarker-guided treatment

The development of biomarker-guided therapeutic strategies represents a central component of precision medicine in autoimmune and inflammatory diseases. Biomarkers provide measurable indicators of biological processes, disease activity, and therapeutic response, thereby enabling clinicians to tailor treatment strategies according to individual patient characteristics (259). In MN, the identification of disease-specific molecular markers has significantly improved diagnostic accuracy and has provided important insights into disease mechanisms and prognosis. One of the most significant advances in the clinical management of MN has been the identification of circulating autoantibodies targeting the M-type phospholipase A2 receptor (PLA2R). Anti-PLA2R antibodies are detected in a substantial proportion of patients with primary MN and are strongly associated with disease activity and immunological status (7). Quantitative measurement of these antibodies has been shown to correlate with disease progression and therapeutic response, making anti-PLA2R levels an important biomarker for monitoring disease activity and guiding treatment decisions (55). Declining antibody titers following immunosuppressive therapy often precede clinical remission, whereas persistent or rising antibody levels may indicate ongoing immune activation and increased risk of relapse. In addition to PLA2R autoantibodies, other molecular markers have been investigated for their potential utility in MN diagnosis and patient stratification. For example, antibodies directed against thrombospondin type-1 domain-containing 7A (THSD7A) have been identified in a subset of patients with primary MN who are negative for PLA2R antibodies (260). Detection of these antibodies provides further evidence for the autoimmune nature of the disease and expands the spectrum of biomarkers available for clinical evaluation. Complement activation products have also been explored as potential biomarkers in MN. Elevated levels of complement components and terminal complement complexes have been detected in kidney biopsy samples and circulating biological fluids of affected patients (260). Because complement activation contributes directly to podocyte injury and glomerular inflammation, monitoring complement-related biomarkers may provide insights into disease activity and therapeutic response. Advances in high-throughput molecular profiling technologies have further expanded the range of candidate biomarkers for autoimmune kidney diseases. Transcriptomic and proteomic analyses of kidney tissues and biological fluids have identified numerous molecules associated with immune activation, inflammatory signaling, and podocyte injury (12, 254). These molecular signatures may help define disease subtypes and enable more precise classification of patients according to underlying pathogenic mechanisms. Biomarker-guided treatment strategies may also facilitate the rational application of immunomodulatory therapies. By integrating molecular biomarkers with clinical parameters, clinicians may be able to identify patients who are most likely to benefit from specific therapeutic interventions. Such approaches could help reduce unnecessary exposure to immunosuppressive drugs while optimizing treatment outcomes. Furthermore, molecular biomarkers may provide valuable tools for evaluating the biological effects of emerging therapeutic agents, including natural compounds with immunomodulatory properties. Overall, the integration of biomarker-based diagnostics with systems biology approaches represents an important step toward the implementation of precision medicine in autoimmune kidney diseases. Continued research aimed at identifying and validating molecular biomarkers will be essential for improving disease stratification, guiding therapeutic decisions, and advancing personalized treatment strategies for patients with membranous nephropathy.

### 7.3 AI-assisted syndrome classification

Artificial intelligence (AI) has increasingly been applied to biomedical research and clinical decision-making as a powerful tool for analyzing complex datasets and identifying patterns that may not be readily detectable through traditional analytical methods. In recent years, AI-driven approaches—including machine learning, deep learning, and data-driven predictive modeling—have been utilized to improve disease classification, diagnostic accuracy, and treatment stratification across a wide range of medical conditions (261). These computational approaches are particularly valuable for studying diseases characterized by complex and heterogeneous pathophysiological mechanisms, such as autoimmune and inflammatory disorders. In the context of TCM, syndrome classification—commonly referred to as pattern differentiation represents a fundamental diagnostic framework used to guide therapeutic decision-making. TCM syndromes are traditionally defined by combinations of clinical symptoms, physiological characteristics, and functional imbalances within organ systems. However, the subjective nature of syndrome differentiation and variability among practitioners have historically posed challenges for standardization and reproducibility in clinical research (262). Recent advances in artificial intelligence have provided new opportunities for addressing these challenges. Machine learning algorithms can analyze large datasets containing clinical symptoms, laboratory findings, imaging results, and molecular biomarkers in order to identify reproducible patterns associated with specific disease phenotypes or syndrome classifications (263). By integrating diverse clinical and biological data sources, AI-based models may facilitate more objective and standardized classification of disease subtypes and clinical phenotypes. In autoimmune kidney diseases such as membranous nephropathy, the application of AI-assisted analytical approaches may enable more precise characterization of disease heterogeneity. For example, machine learning models trained on multi-dimensional datasets—including clinical parameters, serological biomarkers, transcriptomic profiles, and histopathological features—can identify clusters of patients with distinct molecular signatures and disease trajectories (264). Such analyses may improve understanding of disease mechanisms and help guide personalized treatment strategies. AI-based analytical frameworks may also support the integration of traditional medical concepts with modern biomedical data. By analyzing correlations between clinical symptom patterns, molecular biomarkers, and disease outcomes, computational models can help explore potential relationships between traditional syndrome classifications and measurable biological processes (265). These integrative approaches may contribute to the development of more objective diagnostic frameworks that bridge traditional medical knowledge with contemporary systems biology. Furthermore, AI-assisted classification systems may enhance the discovery of therapeutic targets and facilitate drug development. Machine learning models can analyze large-scale pharmacological datasets to identify associations between bioactive compounds, molecular targets, and disease-related signaling pathways (266). Such computational approaches may help identify candidate compounds with potential immunomodulatory effects and predict their interactions with biological networks relevant to autoimmune kidney diseases. Overall, the integration of artificial intelligence with systems biology and precision medicine frameworks provides promising opportunities for improving disease classification, biomarker discovery, and therapeutic development. Continued advances in computational methods and data integration strategies may support the development of more precise and personalized approaches to diagnosing and treating complex immune-mediated diseases such as MN (267).

### 7.4 Network pharmacology and computational drug discovery

Network pharmacology has emerged as an important interdisciplinary framework for understanding how bioactive compounds interact with complex biological systems. Unlike conventional pharmacological paradigms that typically focus on single drug–single target interactions, network pharmacology emphasizes the modulation of multiple molecular targets and signaling pathways within biological networks (50). This approach is particularly relevant for the investigation of natural compounds derived from traditional medicinal plants, which often exert pharmacological effects through simultaneous regulation of several interconnected molecular pathways. The conceptual foundation of network pharmacology integrates systems biology, bioinformatics, and computational modeling to analyze the interactions between bioactive compounds, molecular targets, and disease-associated signaling pathways. By constructing networks that link compounds to proteins, genes, and biological pathways, researchers can identify key regulatory nodes that may represent potential therapeutic targets (268). Such network-based approaches are especially valuable in complex diseases such as MN, where multiple biological processes including immune dysregulation, complement activation, oxidative stress, and podocyte injury interact to drive disease progression. In recent years, computational tools have been increasingly applied to identify molecular targets of natural compounds and predict their pharmacological effects. Databases containing information on chemical structures, protein targets, and signaling pathways enable researchers to perform compound–target network analyses, protein–protein interaction mapping, and pathway enrichment analysis (269). These methods can reveal potential molecular mechanisms through which bioactive compounds influence disease-related biological networks. For compounds discussed in this review, such computational analyses may help clarify how individual molecules interact with immune and inflammatory signaling pathways implicated in MN. For example, network-based analyses have suggested that compounds such as icariin and astragaloside IV may influence signaling networks involving NF-κB, PI3K/Akt, and MAPK pathways, which are central regulators of immune activation and inflammatory responses (52). Similarly, cordycepin has been associated with regulatory networks involving AMPK signaling and inflammasome activation. By mapping these interactions within molecular networks, network pharmacology provides a systems-level perspective on how natural compounds may influence complex disease mechanisms. Another important component of computational drug discovery involves molecular docking and structural bioinformatics. Molecular docking simulations allow researchers to predict interactions between small molecules and protein targets by estimating binding affinity and structural compatibility at the molecular level (270). These computational approaches can provide valuable insights into potential mechanisms of action and help prioritize candidate compounds for experimental validation. When combined with network pharmacology analyses, molecular docking can support the identification of biologically relevant compound–target interactions that may contribute to therapeutic effects.

Artificial intelligence and machine learning techniques are also increasingly integrated into computational drug discovery pipelines. AI-driven algorithms can analyze large chemical libraries and biological datasets to predict pharmacological activity, identify novel drug targets, and optimize candidate compounds for further development (266). Such approaches have accelerated the discovery of bioactive molecules and facilitated the identification of compounds capable of modulating complex disease-associated signaling networks. Importantly, the integration of network pharmacology with experimental pharmacology can provide a powerful strategy for investigating natural compounds derived from traditional medicinal systems. Computational predictions can guide experimental studies aimed at validating molecular targets, signaling pathways, and biological effects. This iterative process between computational modeling and experimental validation may enhance the efficiency of drug discovery and improve understanding of the molecular mechanisms underlying natural product pharmacology. Overall, network pharmacology and computational drug discovery provide valuable methodological tools for investigating the multi-target pharmacological actions of natural compounds. By integrating chemical, molecular, and systems-level data, these approaches may facilitate the identification of novel therapeutic strategies targeting complex immune-mediated diseases such as MN (271).

## 8 Future perspectives

Despite significant progress in understanding the immunological mechanisms underlying MN, important gaps remain in translating mechanistic insights into effective therapeutic strategies. While preclinical studies have provided valuable evidence regarding immune dysregulation, complement activation, and podocyte injury in MN, many aspects of disease pathogenesis and therapeutic intervention require further investigation. In particular, translating findings from experimental models and molecular pharmacology studies into clinically validated therapeutic approaches remains a major challenge. Recent advances in systems biology, biomarker discovery, and computational pharmacology have expanded opportunities for identifying novel therapeutic targets and evaluating candidate compounds derived from natural products. However, the clinical applicability of many immunomodulatory compounds remains insufficiently explored. Future research should therefore prioritize rigorous experimental validation and clinical investigation to determine whether the molecular mechanisms identified in preclinical studies translate into measurable clinical benefits for patients with MN.

### 8.1 Need for mechanistic clinical trials

Most studies investigating bioactive compounds derived from traditional medicinal plants have been conducted in cellular systems or experimental animal models. Although these studies have demonstrated important anti-inflammatory, antioxidant, and immunomodulatory properties, the clinical efficacy and safety of these compounds in human patients with immune-mediated kidney diseases remain largely unexplored (43). Consequently, well-designed clinical trials are necessary to evaluate whether these pharmacological effects translate into meaningful therapeutic outcomes. In addition to evaluating clinical efficacy, future trials should incorporate mechanistic endpoints that allow researchers to assess the biological effects of candidate therapies on disease-related molecular pathways. Such mechanistic clinical trials integrate traditional clinical outcome measures—such as proteinuria reduction, renal function preservation, and disease remission—with molecular biomarkers reflecting underlying pathophysiological processes (272). Examples of mechanistic biomarkers in MN include circulating anti-PLA2R antibody levels, complement activation markers, inflammatory cytokine profiles, and transcriptomic signatures associated with immune activation.

The incorporation of biomarker-based endpoints may provide important insights into how therapeutic agents influence disease mechanisms at the molecular level. For example, monitoring changes in anti-PLA2R antibody titers during treatment may help determine whether a candidate compound effectively suppresses autoreactive B-cell responses. Similarly, evaluating complement activation markers and inflammatory signaling pathways may reveal whether therapeutic interventions reduce immune-mediated injury within the glomerulus. Mechanistic clinical trials may also facilitate the identification of patient subgroups that are more likely to respond to specific therapeutic strategies. Because MN exhibits considerable heterogeneity in terms of immunological mechanisms and disease progression, stratifying patients according to molecular biomarkers or immunological profiles may improve the precision of therapeutic interventions (55). Such approaches align with the broader goals of precision medicine, which aims to tailor treatment strategies according to individual biological characteristics. Furthermore, integrating mechanistic analyses with clinical trials may help clarify the pharmacological actions of natural compounds and support their rational development as therapeutic agents. Combining clinical outcomes with molecular biomarker analysis, transcriptomic profiling, and immunological monitoring could provide comprehensive insight into how candidate compounds modulate immune signaling pathways relevant to MN pathogenesis.

Overall, the development of mechanistic clinical trials represents an important step toward translating molecular discoveries into clinically meaningful therapies. Rigorous evaluation of candidate compounds in well-designed clinical studies will be essential for determining their safety, therapeutic efficacy, and potential role in the management of immune-mediated kidney diseases such as membranous nephropathy.

### 8.2 Drug discovery from TCM compounds

Natural products have historically played a central role in the discovery and development of therapeutic agents. A substantial proportion of modern pharmaceuticals have been derived directly or indirectly from natural compounds, highlighting the importance of natural product libraries as sources of chemically diverse bioactive molecules (42). TCM, which encompasses a vast collection of medicinal plants and bioactive constituents, represents a particularly valuable reservoir for identifying candidate compounds with potential therapeutic relevance for complex diseases. Advances in natural product chemistry and pharmacological screening have enabled the identification of numerous bioactive compounds derived from TCM that exhibit immunomodulatory, anti-inflammatory, and cytoprotective properties. Compounds such as icariin, astragaloside IV, catalpol, and cordycepin illustrate how structurally defined molecules isolated from medicinal plants can serve as lead compounds for drug discovery and development. These molecules possess well-characterized chemical structures and have demonstrated experimentally validated biological effects on signaling pathways involved in immune regulation and inflammatory responses. The process of drug discovery from natural compounds typically begins with the identification of molecules that exhibit desirable biological activity in experimental models. Once candidate compounds have been identified, structure–activity relationship (SAR) analyses can be performed to determine which structural features contribute to their pharmacological properties (273). Through systematic chemical modification and synthesis of derivative molecules, researchers can optimize the biological activity, selectivity, and stability of candidate compounds. Modern drug discovery strategies increasingly incorporate structure-based drug design, which utilizes structural information about target proteins to guide the development of molecules with improved binding affinity and pharmacological efficacy. High-resolution structural techniques, including X-ray crystallography and cryo-electron microscopy, allow detailed characterization of protein–ligand interactions and facilitate rational design of improved therapeutic agents (274). When combined with computational modeling and molecular docking approaches, these techniques can accelerate the identification and optimization of candidate compounds derived from natural products. Another important aspect of drug development involves improving the pharmacokinetic properties of candidate molecules. Many natural compounds exhibit promising biological activity but may have limitations related to poor solubility, limited bioavailability, or rapid metabolic degradation. Pharmaceutical strategies such as chemical modification, nanoparticle-based delivery systems, and prodrug design can be employed to enhance the stability and bioavailability of natural compounds while maintaining their pharmacological activity (275). In the context of autoimmune kidney diseases such as membranous nephropathy, the identification of compounds capable of modulating immune signaling pathways represents an important avenue for therapeutic development. Natural compounds that regulate inflammatory pathways, complement activation, and oxidative stress responses may serve as starting points for the development of new immunomodulatory agents targeting disease-relevant mechanisms. By integrating natural product chemistry with modern pharmacological and computational approaches, researchers can systematically evaluate candidate compounds and optimize them for clinical application.

Overall, TCM-derived compounds represent an important source of structurally diverse molecules with potential therapeutic relevance for immune-mediated diseases. Continued investigation of these compounds using modern drug discovery technologies may facilitate the development of novel pharmacological agents capable of modulating complex molecular networks involved in autoimmune kidney disease.

### 8.3 Integration with immunotherapy

The management of MN has evolved significantly in recent years with the introduction of targeted immunotherapies designed to suppress autoreactive immune responses and reduce immune-mediated injury to the glomerulus. Current therapeutic strategies primarily aim to control the underlying autoimmune process by reducing autoantibody production, modulating immune cell activity, and preventing complement-mediated podocyte damage (35). Agents such as B-cell–depleting monoclonal antibodies, calcineurin inhibitors, and alkylating agents are widely used in clinical practice, although these treatments are associated with potential adverse effects and variable patient responses. Among the most important advances in MN therapy has been the use of B-cell–targeted therapies, particularly the anti-CD20 monoclonal antibody rituximab. Rituximab depletes circulating B lymphocytes, thereby reducing the production of pathogenic autoantibodies such as anti-PLA2R antibodies (33). Clinical trials have demonstrated that rituximab can induce immunological remission and reduce proteinuria in many patients with primary MN. However, not all patients respond to B-cell depletion therapy, indicating that additional pathogenic pathways may contribute to disease progression. Complement inhibition has also emerged as a potential therapeutic strategy for immune-mediated kidney diseases. Because complement activation plays a central role in podocyte injury and glomerular inflammation in MN, targeting complement pathways may help reduce inflammatory damage within renal tissues (12). Several complement inhibitors—including agents targeting C5 activation and downstream components of the terminal complement cascade—are currently being investigated in clinical studies of glomerular diseases. In this context, bioactive compounds derived from traditional medicinal plants may offer complementary mechanisms that could enhance the efficacy of existing immunotherapies. As discussed in earlier sections, several TCM-derived compounds regulate molecular pathways involved in inflammatory signaling, immune cell activation, oxidative stress responses, and metabolic regulation. These mechanisms may intersect with pathways targeted by conventional immunotherapies, suggesting the possibility of combinational therapeutic strategies. For example, compounds that suppress NF-κB–mediated inflammatory signaling or inhibit NLRP3 inflammasome activation may reduce inflammatory amplification downstream of immune complex deposition and complement activation. Similarly, compounds that enhance antioxidant defenses or protect podocytes from mitochondrial dysfunction may help preserve glomerular structure while immunotherapies address upstream autoimmune processes. By targeting complementary aspects of disease pathogenesis, combined therapeutic approaches may improve overall treatment outcomes. The integration of natural immunomodulatory compounds with modern immunotherapies may also support strategies aimed at reducing treatment-related toxicity. Because conventional immunosuppressive therapies can increase susceptibility to infection and other adverse effects, adjunctive agents capable of modulating inflammatory pathways without broadly suppressing immune function may allow lower doses of immunosuppressive drugs to be used while maintaining therapeutic efficacy (246). However, rigorous experimental and clinical studies are necessary to evaluate potential interactions between natural compounds and established immunotherapeutic agents. Future research should therefore investigate the pharmacological compatibility and potential synergistic effects between TCM-derived compounds and existing immunotherapies. Such studies may include mechanistic investigations of molecular pathway interactions, pharmacokinetic analyses, and carefully designed clinical trials evaluating combined therapeutic strategies. By integrating insights from immunology, pharmacology, and systems biology, these approaches may contribute to the development of more effective and personalized therapeutic strategies for patients with membranous nephropathy.

## 9 Conclusion

Membranous nephropathy (MN) is a prototypical immune-mediated glomerular disease characterized by the formation of autoantibodies against podocyte antigens, immune complex deposition along the glomerular basement membrane, complement activation, and progressive podocyte injury. Advances in molecular immunology have substantially improved the understanding of MN pathogenesis, particularly with the identification of target antigens such as phospholipase A2 receptor (PLA2R) and the recognition of complement-mediated mechanisms contributing to glomerular damage. Despite these advances, current therapeutic strategies remain limited by incomplete response rates, treatment-associated toxicity, and variability in patient outcomes. In recent years, increasing attention has been directed toward bioactive compounds derived from traditional medicinal plants as potential modulators of immune and inflammatory pathways. Several compounds isolated from TCM including icariin, astragaloside IV, catalpol, cordycepin, and Lycium barbarum polysaccharides possess defined chemical structures and have demonstrated experimentally validated immunomodulatory effects in preclinical studies. These compounds regulate key molecular pathways involved in inflammatory signaling, oxidative stress responses, immune cell activation, and metabolic regulation. Because these pathways contribute directly to podocyte injury and immune dysregulation in MN, natural compounds capable of modulating these signaling networks may represent promising candidates for further therapeutic investigation. The integration of systems biology, multi-omics technologies, biomarker-based diagnostics, and computational pharmacology provides powerful tools for advancing the discovery and development of novel therapeutic strategies. Approaches such as network pharmacology and artificial intelligence–assisted analysis enable comprehensive exploration of complex biological interactions and facilitate the identification of candidate compounds capable of targeting multiple disease-associated pathways. Such integrative strategies may accelerate the translation of natural product pharmacology into clinically relevant therapeutic applications. Future research should prioritize mechanistic clinical trials, biomarker-guided patient stratification, and systematic investigation of compound–target interactions using modern pharmacological and computational approaches. In addition, evaluating potential synergy between natural immunomodulatory compounds and established immunotherapies may provide opportunities to enhance treatment efficacy while minimizing adverse effects. Overall, the integration of traditional medicinal knowledge with contemporary molecular immunology and systems biology offers a promising framework for exploring novel therapeutic strategies in immune-mediated kidney diseases. Continued interdisciplinary research may contribute to the development of more effective and personalized treatment approaches for patients with membranous nephropathy.
